# An interpretable framework for gastric cancer classification using multi-channel attention mechanisms and transfer learning approach on histopathology images

**DOI:** 10.1038/s41598-025-97256-0

**Published:** 2025-04-16

**Authors:** Muhammad Zubair, Muhammad Owais, Taimur Hassan, Malika Bendechache, Muzammil Hussain, Irfan Hussain, Naoufel Werghi

**Affiliations:** 1 Interdisciplinary Research Center for Finance and Digital Economy, King Fahd University of Petroleum and Minerals, 31261 Dhahran, Saudi Arabia; 2 Department of Mechanical & Nuclear Engineering, Khalifa University, Abu Dhabi, United Arab Emirates; 3 Departement of Electrical and Computer Engineering, Abu Dhabi University, Abu Dhabi, United Arab Emirates; 4 ADAPT Research Centre, School of Computer Science, University of Galway, H91 TK33 Galway, Ireland; 5 Department of Software Engineering, Faculty of Information Technology, Al-Ahliyya Amman University, Amman, Jordan; 6 Department of Computer Science, Khalifa University, Abu Dhabi, United Arab Emirates

**Keywords:** Cancer, Cell biology, Computational biology and bioinformatics, Biomarkers

## Abstract

The importance of gastric cancer (GC) and the role of deep learning techniques in categorizing GC histopathology images have recently increased. Identifying the drawbacks of traditional deep learning models, including lack of interpretability, inability to capture complex patterns, lack of adaptability, and sensitivity to noise. A multi-channel attention mechanism-based framework is proposed that can overcome the limitations of conventional deep learning models by dynamically focusing on relevant features, enhancing extraction, and capturing complex relationships in medical data. The proposed framework uses three different attention mechanism channels and convolutional neural networks to extract multichannel features during the classification process. The proposed framework’s strong performance is confirmed by competitive experiments conducted on a publicly available Gastric Histopathology Sub-size Image Database, which yielded remarkable classification accuracies of 99.07% and 98.48% on the validation and testing sets, respectively. Additionally, on the HCRF dataset, the framework achieved high classification accuracy of 99.84% and 99.65% on the validation and testing sets, respectively. The effectiveness and interchangeability of the three channels are further confirmed by ablation and interchangeability experiments, highlighting the remarkable performance of the framework in GC histopathological image classification tasks. This offers an advanced and pragmatic artificial intelligence solution that addresses challenges posed by unique medical image characteristics for intricate image analysis. The proposed approach in artificial intelligence medical engineering demonstrates significant potential for enhancing diagnostic precision by achieving high classification accuracy and treatment outcomes.

## Introduction

Gastric cancer (GC), or stomach cancer, is a formidable health challenge with significant global implications. It has a long-standing history and remains one of the most prevalent and deadly cancers worldwide^1^. Early detection and timely treatment are crucial factors in improving patient outcomes and reducing mortality rates associated with this disease. Recently, an increasing focus has been on understanding GC’s epidemiology, risk factors, and biological characteristics. Such knowledge contributes to developing effective prevention strategies, diagnostic approaches, and treatment modalities. GC’s prevalence, morbidity, and mortality rates necessitate continuous efforts to improve detection methods and ensure early intervention for optimal patient care. According to recent statistics, GC has become the fifth most prevalent disease globally and the fourth leading cause of death, making it a significant public health concern^2,3^. It is responsible for many cancer-related deaths, ranking as the third leading cause of cancer mortality worldwide^3^. These statistics emphasize the urgent need for improved detection and management strategies to address the impact of GC on global health.

The current diagnostic methods for GC mainly involve endoscopic examinations, biopsies, and histopathological analysis. Endoscopy allows for direct visualization and tissue sampling, enabling clinicians to identify suspicious lesions and collect biopsy samples for further analysis. Tissue staining techniques employed for the examination of anatomical connectivity^4–6^, cancer progression^7^, forensic pathology^8^, studying tissue morphology^9,10^, disease surveillance^11^, and genetic alterations^12,13^ Other applications of immunohistochemistry staining are discussed in detail^14^.

The histopathological study of GC constitutes the gold standard for identifying GC^15^. The diagnosis of GC is mainly through pathological biopsy, which is stained with hematoxylin and eosin (H&E). The histopathological examination provides crucial information about tumor characteristics, including histological type, grade, and stage. The nucleus and cytoplasm of tissue sections are examined by viewing the H&E stained sections, highlighting the fine structure of cells and tissues for physician observation.

However, these diagnostic approaches have limitations, including invasiveness, sampling errors, and interobserver variability, which may impact diagnostic accuracy^16^. Under a microscope, the biopsy’s morphology and tissue properties are scrutinized, and the doctor’s expertise is synthesized to determine the detection findings. Nonetheless, individual pathology professionals rely on their own experiences and contextual circumstances when making diagnoses, potentially leading to discrepancies in their interpretations of tissue pathology images. Additionally, pathologists are responsible for analyzing numerous histology images regularly. Maintaining continuous focus and working extended hours may increase the probability of professionals making diagnostic errors. Consequently, precise pathologist detection of stomach cancer is a significant issue^17^. In addition, early diagnosis is paramount in achieving favorable outcomes for GC patients. Detection at an early stage allows for more effective treatment options, including curative surgery, and can significantly improve survival rates. Therefore, developing reliable, accurate, and sensitive screening and diagnostic methods is essential to guarantee GC’s accurate and early detection.

The above-mentioned problems could be addressed by introducing a computer-aided diagnosis (CAD) system that could identify pathological images of GC to alleviate the lack of pathologists and lower the incidence of histological examination misdiagnosis^18^. Advanced algorithms could be developed to help shorten the processing time and allow the CAD system to make objective decisions^19–22^, classification^23–29^, and segmentation^30^ during cervical cancer^31,32^, skin cancer^33^, and neurological disorders^4,34^ detection. In the past, the rapid development of CAD technology for GC, which can more rapidly and reliably identify cancer locations, has been made possible by the constant advancements in image processing, machine learning (ML), and pattern recognition algorithms^1,35,36^. These algorithms utilize ML and deep learning (DL) techniques to analyze diagnostic data, such as imaging, biomarkers, and clinical parameters. Although these algorithms promise to improve diagnostic accuracy, they also have limitations. Factors such as dataset heterogeneity, lack of standardization, and interpretability of results may hinder their widespread implementation in clinical practice^37,38^. Moreover, the conventional ML techniques used in traditional CAD^19,24^ approaches operate as follows: First, the manual extraction of visual attributes, including form, color, and texture. Afterwards, a classifier categorizes the retrieved characteristics^39^. Convolutional neural network (CNN) models allow for automatic feature learning in computers, replacing the subjectivity of feature extraction in ML. This has significantly improved the accuracy and effectiveness of CAD^20–22,40^. The drawback of CNN models is that they do not effectively extract reliable data from small datasets. Because of this limitation, it is crucial to integrate CNN models with an attention mechanism.

Recent studies in GC classification using histopathological images have two major challenges, including the lack of interpretability of the models and the limited generalizability of the data sets. Interpretability is crucial for clinical adoption to gain trust of the clinicians in model prediction. Although some studies have incorporated attention mechanisms^41^, they do not provide proper visualization of the decision-making process. To address this, we integrate Grad-CAM visualizations within our multi-channel attention-based framework, enhancing model transparency. In addition, heterogeneity of the dataset poses a significant challenge due to variations in staining techniques, scanner types, and demographic differences between medical centers. Traditional models often struggle to generalize well under these conditions. Our approach mitigates this issue by using a multi-scale feature extraction mechanism and a transfer learning-based pipeline^42^ trained on diverse histopathology GasHisSDB and HCRF datasets. This enhances the model’s adaptability to different clinical environments. These contributions fill critical gaps in the literature, providing a more interpretable and robust framework for GC classification.

### Attention mechanism

According to cognitive research, humans only take in a small portion of all observable information due to processing bottlenecks. Inspired by the human visual system, attention mechanisms are techniques for directing focus to the most crucial picture areas while ignoring irrelevant ones^43^. It prioritizes the most informative signal component while allocating computing resources^44^. Researchers searched for a model of visual selective attention to mimic how people perceive visual information, model how people’s attention is distributed when viewing still images and moving pictures, and broaden the model’s usefulness. Attention methods have been shown to enhance model performance and are also congruent with the perceptual process of the human brain and eyes. Most research integrating DL with visual attention processes in computer vision, for instance, focuses on using masks. According to the masking concept, a new layer with a new weight is used to identify the essential characteristics in the image data. DNNs may develop attention by learning and training the portions of each new image that require attention. As attention processes have developed into several categories throughout development, different models stress distinct feature domains. These models are used for various tasks, including classification, detection, segmentation, model improvement, video processing, and more. Attention mechanisms can be categorized into channel attention, spatial attention, mixed attention, and self-attention. Channel attention approaches, some typical works of which include the aforementioned Squeeze-and-Excitation Network (SENet)^45^, Efficient Channel Attention (ECANet)^46^, and Style-based Recalibration Module (SRM)^47^ produce attention mask throughout the channel domain and utilize it to pick significant channels. Spatial Transformer Networks (STN)^48^ and Gather-excite Networks (GENet)^49^ are two examples of spatial attention approaches that produce attention masks across geographic domains and utilize them to pick significant spatial locations. Convolutional block attention module (CBAM)^50^ and coordinate attention^51^ are examples of channel and spatial attention techniques that combine the benefits of both to create 3-D attention maps. Some other recent techniques concentrating on branch and temporal attention results were proposed^52,53^.

### Attention mechanism enhanced CNN

CNN’s performance has been accelerated by attention processes, sparking excellence across a range of visual difficulties, including classification, detection, segmentation, model improvement, video mastering, and more^54^. Attention techniques often take the form of plug-and-play attention modules that may enhance a block’s convolutional outputs and help the entire network learn more illuminating information^55^. Due to the integration of attention modules in some advanced CNN designs, such as the SE module added to MobileNet V3, that network version performs better than MobileNet V1 and MobileNet V2^56^. To overcome challenges like complex backdrops, dispersed lesions, and inter-class resemblances - think abnormality detection and normal cell identification-researchers in the field of image classification are increasingly incorporating attention modules into their custom network designs^57–62^. However, building these attention modules frequently entails complex elements, such as pooling options, which might add parameters and computational load and be unwelcoming for lightweight network topologies.

**Fig. 1:**
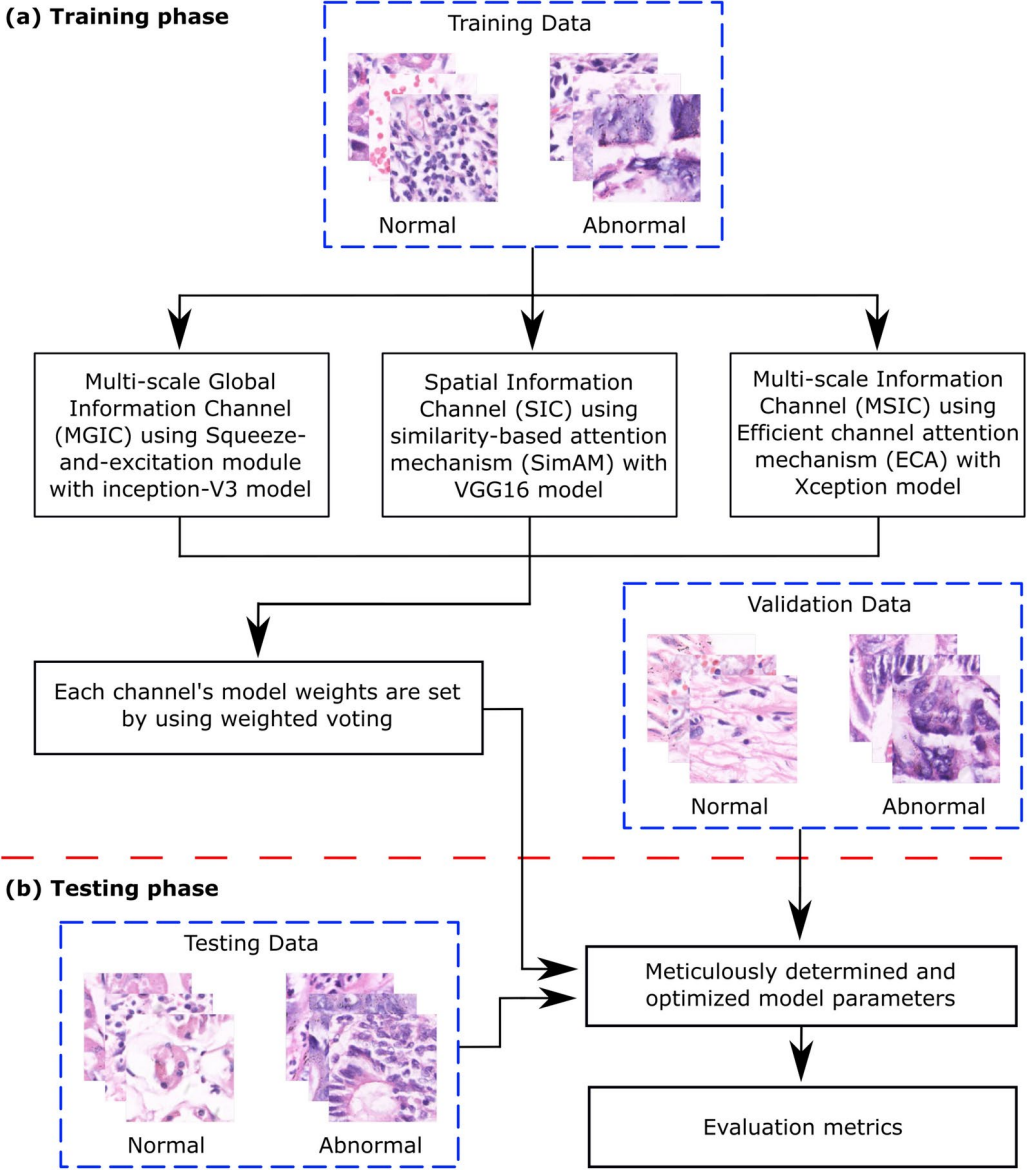
A general overview of the proposed methodology. Dotted red line separates (**a**) training and (**a**) testing phase.

Our research provides a unique strategy to address the intrinsic complexity of medical picture information, where complex components and restricted inequalities across several phases make it difficult to identify relevant attention areas using a single AM. In particular, this study provides a learning paradigm that uses a multi-channel attention mechanism (MCAM). Our proposed framework improves the accuracy of GC histopathology image classification procedures by overcoming the challenges posed by complex medical pictures. The flow chart of the proposed framework is visually depicted in Fig. 1. The methodology has two phases: training and testing. The MCAM model, which consists of three channels: multi-scale global information channel (MGIC), spatial information channel (SIC), and multi-scale spatial data channel (MSIC), is used for learning. After numerous epochs, the weighted voting technique is used to extract the model parameters of the learning from the MCAM model using the training pictures. The test photos are provided while maintaining the optimized model parameters to achieve the GC histopathological image classification task results. The model parameters are finally kept to achieve the GC histopathological image classification job results, and the test pictures are input.

The main contributions of this research study are as follows:

- A multi-channel attention mechanism (MCAM)-based framework using transfer learning (TL) is introduced as an efficient GC classifier. Three channels, including multi-scale global information channel (MGIC), spatial information channel (SIC), and multi-scale spatial information channel (MSIC) using attention mechanism could extract comprehensive multi-scale local, global, and spatial information, are integrated and deployed with TL, resulting in an effective classification approach.
- The reliability of the proposed MCAM model is underscored by its consistent performance across two distinct datasets, highlighting the model’s inherent robustness.
- The proposed model has achieved the highest evaluation metrics compared to the conventional deep learning approaches and previously existing competitive studies on GC classification using histopathology images.
- The growing need for transparent AI tools in medical diagnostics is met by including attention mechanisms and strengthening model interpretability. The regions of interest are depicted using Grad-CAM visuals, which promote therapeutic confidence and provide insights into the decision-making process. A comparative analysis with cutting-edge deep learning models, including VGG-16, Xception, Vision Transformers (ViT), and ensemble approaches, highlights the superior performance of the proposed MCAM framework.

To improve the classification of gastric cancer histopathology images, the hypothesis was to test an MCAM-based framework that may overcome the limitations of conventional deep learning models through enhanced feature extraction, dynamically focusing on relevant features and capturing intricate relationships in medical data. The study offers a thorough and efficient solution for GC classification by tackling dataset heterogeneity, interpretability issues, and the lack of robustness in earlier approaches. The paradigm differs from other approaches in the field as it incorporates MCAM, transfer learning, and a focus on interpretability.

## Related work

We undertake two explorations in this section. First, a full and in-depth description of deep learning techniques is presented, exploring their fundamental ideas and wide range of uses. Next, we focus on a comprehensive analysis of GC identification and categorization using a thorough investigation of the DL techniques utilized in previous competitive research. GC detection and classification are two areas in which this two-pronged approach seeks to provide the reader with a deep understanding of DL techniques and a nuanced understanding of their particular applications.

### Overview of deep learning methods

CNN models are the most popular DL techniques used in computer vision tasks. Transformer and multilayer perceptron (MLP) models have also gained popularity because of their constant improvement. Particularly, many biological image analysis tasks, such as histological image analysis^63–67^, cytopathological image analysis^68–71^, microorganism image analysis^72–74^, COVID-19 identification^75,76^, and sperm image analysis^77,78^, make extensive use of DL techniques. These models can translate low-level aspects of the data into high-level abstract features. This trait makes DL models stronger than shallow ML models in feature representation^79,80^. The ongoing advancements in CNN models specifically address three main areas: the network’s depth, width, and a hybrid combination of both^81,82^. The ResNet^83^, VGG^84^, and DenseNet^85^ models boost the network depth by employing small convolutional layers, dense layers, and residual mechanisms to enhance model performance. The Xception^86^ and Inception-V3^87^ models boost the network width by using separable convolutional blocks and multi-scale inception blocks. Some models, such as ResNeXt^88^ and InceptionResNet^89^, efficiently combine residual mechanisms and inception blocks during the feature extraction. InceptionResNet increases network depth and width. Consequently, classification performance is significantly improved, representing an important breakthrough in network optimization.

In the contemporary landscape of AI research, transformer models^90^, are finding promising applications in unraveling complex challenges within computer vision. Transformer models categorically unfold into two primary factions: a fusion with convolutional neural networks (CNN) and the realm of pure, unadulterated transformer models^91^. Pure transformer models include ViT^92^, CaiT^93^, DeiT^94^, and T2T-ViT^95^ models. Transformer models combined with CNNs are the CoaT^96^, LeViT^97^, and BoTNet^98^ models, which input the feature maps created by convolution of images into the transformer encoder. MLP models are enhanced versions of transformer models and are improved by substituting the self-attention layers of the ViT^92^ model with multiple perceptions.

### Gastric cancer detection using deep learning methods

DL is a type of ML that can identify more abstract information from input data over time^99–101^. DL has recently caught oncologists’ interest. Oncology has seen significant advancements in DL, which is superior to traditional ML methods^102,103^. DL on pathology images for the spatial organization and molecular correlation of tumor-infiltrating lymphocytes was presented^104^.

A study^105^ proposed a DL system for evaluating lymph node and tumor locations using whole-slide images. So, DL models could aid pathologists in diagnosing lymph nodes to identify new prognostic markers that are challenging to quantify manually. In a recent study^30^, a Naive Bayes classifier with the Gaussian Mixture Model and a novel, improved Fuzzy c-means clustering algorithm were proposed for improved classification and segmentation, respectively. A binary image segmentation method enables cancer detection at the pixel level by utilizing a CNN of DeepLab v3 architecture^106^. On the used GC dataset, the authors claim that their AI aid system has an average specificity of 0.806 and a sensitivity of 0.996. Another study made a whole-slide gastric histopathology dataset (GasHisSDB) publicly available^67^. In addition, three CNN classifiers, a unique transformer-based classifier, and seven traditional ML classifiers were tested on this dataset^67^. It was found that the accuracy rates of different classifiers differ significantly; the DL’s highest accuracy was 0.965, and its lowest was 0.862. A study^107^ presented an automated method using TensorFlow DL packages to classify tumor type detection by categorizing the GC dataset having whole-slide images. In another study^108^, DL-based models were used to identify tumors and forecast the course of GC by examining pathological images. In a study by^109^, Epstein-Barr virus (EBV)-positive and microsatellite instability (MSI)/mismatch repair deficient (dMMR) tumors were included that used a histology-based DL model to screen for immunotherapy-sensitive subgroups. Likewise, another study^110^ proposed an efficient DL model to detect EBV-associated GC using H&E-stained images. An ensemble model that combines the decision of multiple DL models managed to attain high accuracy for GC detection using histopathology images^41^. The authors justify the improved performance due to important feature extraction, even from the smaller patches. However, the limitations include higher computational costs. Another DL-based ensemble model using H&E-stained images was presented^111^ to identify the Lymphovascular invasion, which is an indirect predictor of GC.^112^ proposed an ensemble approach that combines the capabilities of ResNet50, VGGNet, and ResNet34 that outperforms the models like EfficientNet and ViTNet. The ensemble model achieves promising accuracy as a result of integrating the mentioned models. This demonstrates the effectiveness of ensemble models in capturing key features offering a significant advantage in GC classification. A hybrid DL and gradient-boosting approach has proven highly effective in classifying gastric histopathology images^113^. Grad-CAM visualizations confirm that the model focuses on relevant histological features, enhancing interpretability. The consistent accuracy and robust performance across metrics demonstrate its potential for reliable GC screening. Feature fusion strategies^114^ were used using a support vector machine and random forest to classify the histopathology images for GC classification. Cross-magnification experiments yielded promising results, achieving accuracies of nearly 80% and 90% when tested on unseen images at varying resolutions.

In a study, radiopathomics models were developed using Logistic regression, NaiveBayes, and Support vector machine, integrating pathomics with radiomics features to classify GC stage^115^. A DL-based prediction was made^116^ using primary tumor slide score and histopathological lymph node status. A multimodal fusion DL model was proposed using histopathology images to predict GC tumor mutational level^117^. In short, DL approaches have shown better results in detecting and categorizing GC^118^. However, a significant issue that needs to be resolved is the improvement of assessment metrics further to boost the reliability and robustness of these approaches.

In a related study^119^, a promising approach for the efficient classification of whole-slide images in gastrointestinal pathology was shown by this CNN/RNN combo. The authors classified biopsy histopathology whole-slide images of the stomach and colon into three categories: adenocarcinoma, adenoma, and non-neoplastic, employing CNN and recurrent neural networks (RNNs). To improve the algorithm’s resilience to visual changes and provide a regularization effect, several data augmentation approaches were used in conjunction with the conventional inception-v3 network architecture. As a feature extractor, the trained inception-v3 network provided input to an RNN model that could deal with length sequences and generate a single output. To confirm the methodology, the study used external datasets from the TCGA-STAD and TCGA-COAD programs, which are publically accessible and may be accessed via the Genomic Data Commons portal. The work^120^ addresses issues like label noise and feature aggregation redundancy in multi-instance learning for cancer diagnosis utilizing whole-slide images. Inter-bag discrimination and fine-grained feature encoding are enhanced by the suggested dual-curriculum contrastive MIL technique. Its potential to improve whole-slide image-based cancer prognostic analysis has been demonstrated by experiments performed on public datasets, which demonstrate better performance compared to state-of-the-art techniques. To address the unpredictability and predictive constraints of Laurén classification, a study^121^ developed a DL model for GC classification. The DL model demonstrated great classification performance and superior patient survival stratification compared to pathologists, demonstrating its promise as a diagnostic and prognostic tool. It was trained using TCGA data (N=166) and externally verified on European (N=322) and Japanese (N=243) cohorts. Researchers examined the shortcomings of conventional staining methods, like IHC and EBER-ISH, in precisely distinguishing GC molecular subclasses^122^. To predict molecular subclasses directly from hematoxylin-eosin-stained histology, they utilized an ensemble CNN. The TCGA-based decision tree for GC subtyping was challenged by the model’s identification of intra-tumoral heterogeneity and overlapping subclass traits. A study developed deep learning-based models, GastroMIL and MIL-GC, to assist in diagnosing GC and predicting overall survival using hematoxylin and eosin-stained pathological images^108^. Trained on cohorts from Renmin Hospital of Wuhan University and the Cancer Genome Atlas, with external validation from the National Human Genetic Resources Sharing Service Platform, achieved a diagnostic accuracy of 0.920, comparable to expert pathologists. While the focus of this review is on gastric cancer, it is noteworthy that deep learning-based approaches have also been successfully applied to other types of cancer, including urological cancers. For instance, studies on urology cancers^123^ have demonstrated the effectiveness of AI models in diagnosing, predicting, and treating various subtypes such as prostate^124^, bladder^125^, and renal cancers^126^, with detection accuracies ranging from 77% to 95%.

The motivation for this study arises from the limitations of current GC detection and diagnosis methods, which have primarily relied on traditional ML models. Albeit DL models have shown potential, they still require further refinement to improve their effectiveness. Past investigations highlight that attention mechanisms enhance DL model efficiency, but there is significant untapped potential in using multiple attention mechanisms to extract multi-scale information. Additionally, integrating the attention mechanisms with transfer learning could improve diagnostic efficiency. In the existing literature we have found voids, including extraction of comprehensive multi-scale information and incorporation of multiple attention mechanism for enhanced diagnostic performance in GC. Therefore, this study aims to develop an MCAM framework utilizing a transfer learning approach to create a more robust and efficient automated GC diagnostic system.

## Materials and methods

**Fig. 2:**
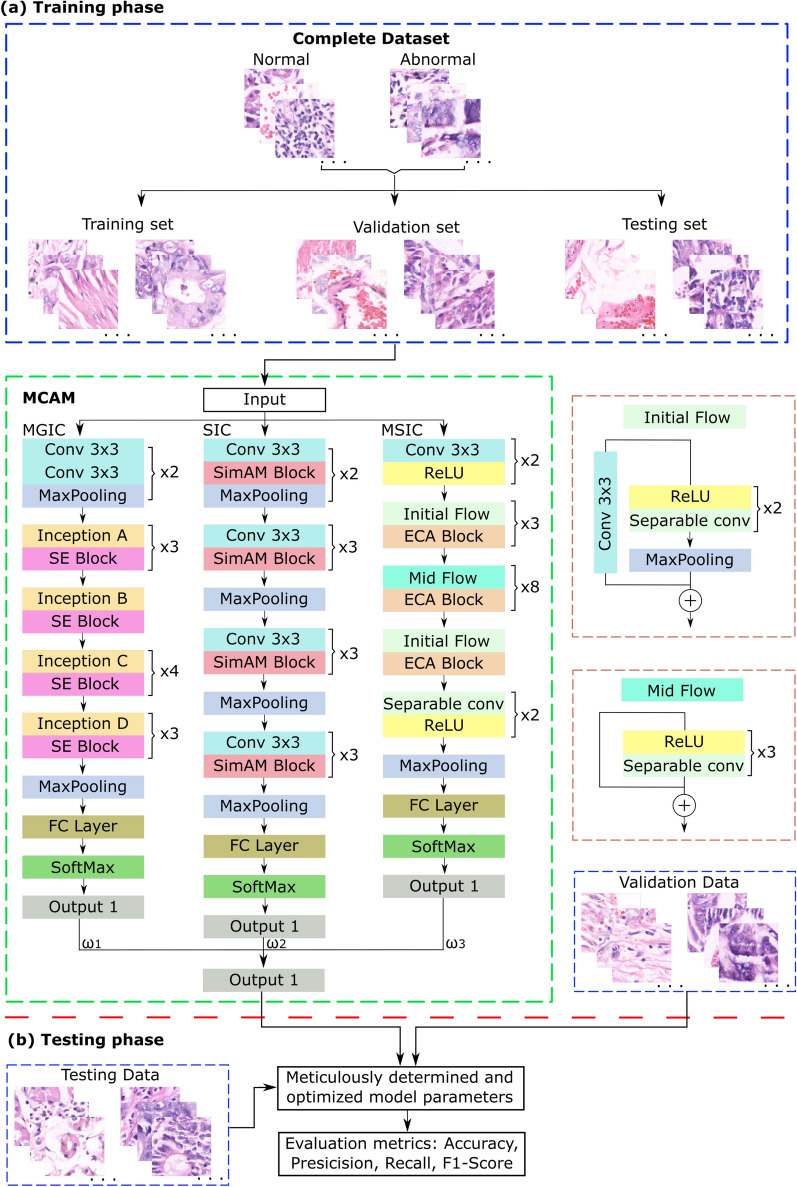
A complete architecture of the proposed methodology having three channels, namely MGIC, SIC, and MSIC, using SE, SimAM, and ECA attention mechanisms with Inception-V3, VGG-16, and Xception CNN models, respectively. The (**a**) training and (**b**) testing phases are separated with red dotted line.

This section delves into an in-depth examination of the three key components fundamental to our suggested framework: TL, attention mechanisms, and CNNs. We believe our detailed explanation of these fundamental components will give readers the knowledge they need to appreciate the subtleties of our suggested framework. The following explanation thoroughly explains the MCAM architecture, as illustrated in Fig. 2. This step-by-step dissection is designed to promote coherent comprehension, guaranteeing that readers can assimilate the framework’s theoretical foundations and architectural nuances in an orderly fashion.

### Convolutional neural network

A CNN is a feedforward neural network distinguished by its distinct design, incorporating convolution and depth computations. CNNs are made up of many layers, each with a distinct function. The convolutional layer, which uses convolution kernels to extract image features, is the main part. The input feature map is then condensed, highlighting key features using the pooling layer. The fully connected layer creates connections between all features and performs classification using a classifier as its last step. The information retrieved by the convolutional layers in the context of CNNs can be divided into two main categories: global and local. The comprehensive representation of an image inside its class is called global information. Local information, often known as spatial information, examines the characteristics of narrow, isolated sections inside the image. Smaller convolution kernels often extract this data type, enabling the network to recognize finer details and localized features essential for classification tasks. In our proposed methodology, we have employed the CNN architectures, including Inception-V3^87^, VGG-16^84^, and Xception^86^. Each has a special layout and set of features. These networks have been extensively employed for various computer vision tasks, including object identification, feature extraction, and image categorization. The specific needs of the task and the available processing resources influence the architecture choice.

### Transfer learning

CNN models require a lot of data and computer power to train from scratch, resulting in lengthy training durations. The training issue is further exacerbated by the peculiarities of medical datasets. TL stands out in this situation as an unprecedented approach to overcoming these difficulties in the field of CAD work^127^. TL is an ML technique that uses a previously trained model for a different job^128^. The TL procedure consists of two parts. The first step is choosing an original dataset and pre-training on it. The second step involves fine-tuning the pre-trained model using the target task’s dataset.

The ImageNet is a widely used dataset with over a million images across 1000 classes for image processing applications^129–131^. The ImageNet dataset, recognized for its extensive and varied collection of images, is the original dataset for pre-training the model in the particular instance covered in the research. However, using the conventional TL technique to pre-train MCAM models directly presents significant difficulties because of limitations in workstation computer capacity. So, we have modified the TL technique to work around our computational constraints. This improved method involves layer-by-layer loading into the MCAM model of the pretraining parameters from conventional CNN models, such as VGG-16, Inception-V3, and Xception, made available through the PyTorch Vision package. The Single Information Channel (SIC), Multi-Global Information Channel (MGIC), and Multi-Scale Spatial Information Channel (MSIC) components, which are described in Fig. 2, are the elements of the MCAM architecture to which these parameters belong. Notably, during training, these loaded layers stay frozen. The completely connected layers and AM layers are at the center of the fine-tuning process, where the model adjusts to the specifics of the target CAD work. Additionally, a weighted voting system provides the channels with the proper weights, ensuring the model successfully incorporates data from each source. This novel approach maximizes the utility of pre-trained models by utilizing the generic feature extraction capabilities of pre-trained models and customizing them to the unique requirements of the CAD task. A compromise has been discovered between utilizing prior information and adapting the model to the specifics of medical picture analysis by combining TL with selective fine-tuning.

### Multi-channel attention mechanism

One of the most critical ideas in the field of DL is the AM method^132^. When only one AM is used, it may be difficult to distinguish between important details and extraneous information, resulting in the decision-making process including extraneous or redundant information. Therefore, the accuracy and effectiveness of the model’s predictions may be jeopardized. Innovative methods, like MCAM, that concentrate on concurrently recording connections across several channels or feature maps, are necessary to overcome these constraints. By doing this, MCAMs improve the model’s capacity to identify important patterns and eliminate superfluous or duplicate data, thereby increasing the precision and dependability of the predictions made by the model. We propose an MCAM model that uses three channels, MGIC, SIC, and MSIC, to extract characteristics from various viewpoints. These three complementing channels improve the accuracy of categorization tasks and the precision of identifying attention areas.

**Fig. 3:**
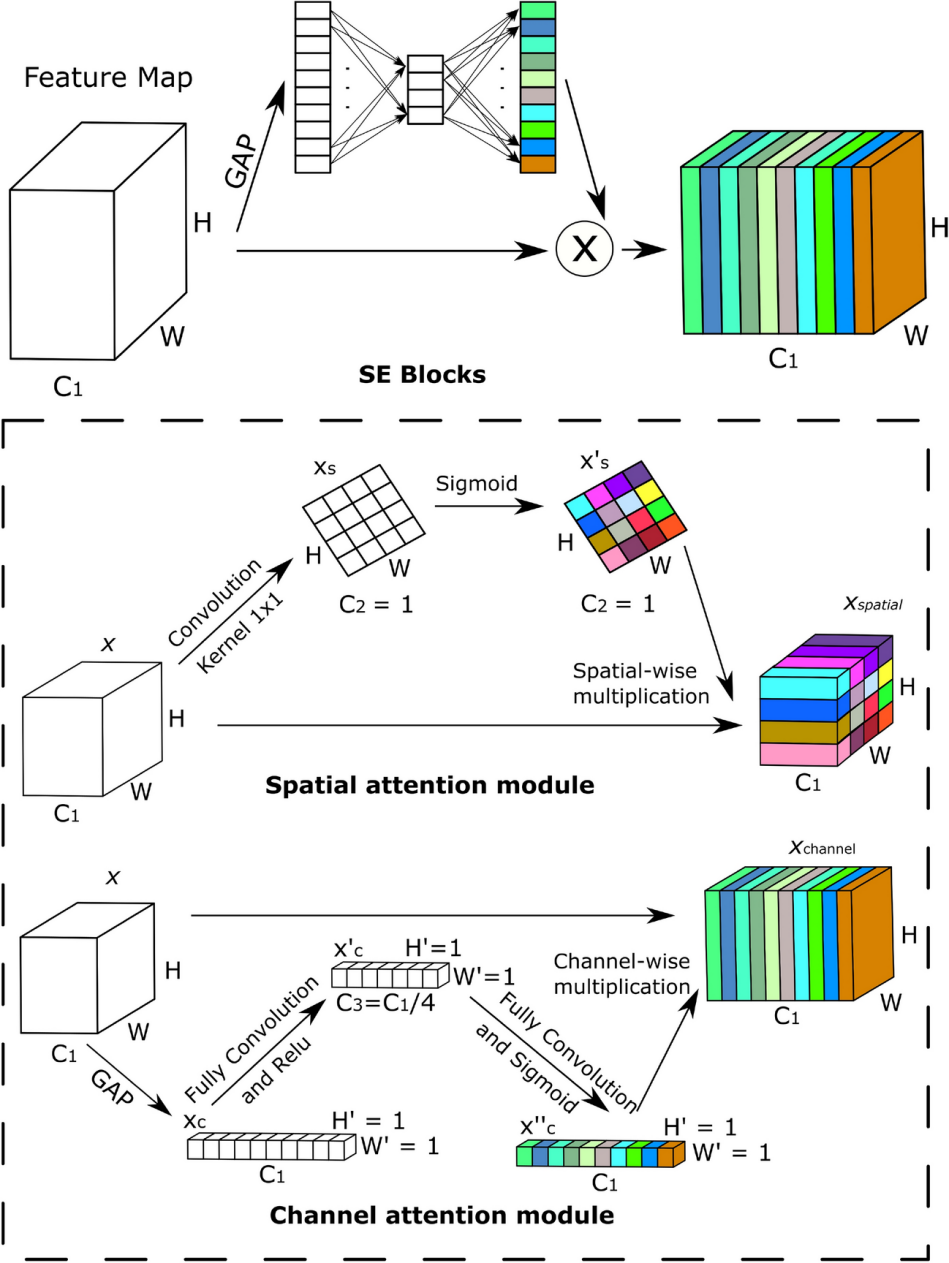
Structure of squeeze-and-excitation (SE) module after each inception block in multi-scale global information channel (MGIC). The outlined border shows the structure of the spatial and channel attention module. H, C, and W represent height, channel, and width, respectively. Abbreviation: GAP stands for global average pooling.

***MGIC:*** The model in the MGIC is contemplated to be able to extract multi-scale global data. The Inception-V3 model^87^, rooted in GoogleNet^133^, is widely regarded as the optimal CNN model for capturing comprehensive global information. The Inception-V3 model employs a distinctive convolution technique, breaking down large filter sizes through parallel and factorized convolution rather than increasing network layers. The term “inception structure” encompasses the entire decomposition module. This model also features five distinct inception structures, each with unique elements. The Inception-V3 model substantially reduces parameters relative to other models by adopting an Inception module instead of a large convolution kernel. Furthermore, it replaces a fully connected layer with a global average pooling (GAP) layer. Because of its parallel convolution structure and partially big convolution kernels, Inception-V3 among CNN models excels at extracting global multi-scale information. Therefore, to extract features from MGIC, the Inception-V3 model is chosen. The Inception-V3 model implements the extraction of multi-scale information by concatenating various sized receptive fields, and each feature map’s channel domain reflects the multi-scale capability of the Inception-V3 model. The MGIC’s SE attention mechanism, which has a good distribution of channel weights, is chosen to increase the weighting of the channel features^45^. The structure of the SE attention mechanism is shown in Fig. 3. Squeeze and excitation are the two stages of the SE attention process. The squeeze phase pools the global averages to encode all spatial features into a single global feature to produce channel-wise statistics. The dimensionality-reduction and dimensionality-increasing layers are two completely connected layers used in the excitation phase to determine the channel-wise importance. The sigmoid activation function then determines the final channel-wise weights. The SE module includes channel and spatial attention modules, as shown in Fig. 3 outlined in the dotted border. The channel and spatial modules help the network learn “what” and “where” to pay attention to the channel and spatial axes. The spatial attention module uses the inter-spatial relations of certain features to produce a spatial attention map. The convolution operation (kernel: [1, 1], stride: [1, 1], channels: 1) is used to obtain $x_{s}$(H x W x 1) from the input *x* (H x W x C). Here, H, W, and C represent height, width, and the channel, respectively. By spatially multiplying the input *x* and the $x_s$, the channel is transformed from $C_1$ to $C_2$, and the spatial attention map $x_{spatial}$ (H x W x $C_1$) is produced. This transformation of $C_1$ to $C_2$ and back to $C_1$ in the spatial attention module is illustrated in Fig. 3 within the outlined border.

The channel attention module creates a channel attention map and can selectively boost helpful features while suppressing invalid ones. A GAP operation on the input x produces $x_c$(1 x 1 x $C_1$). Full convolution (channels: $C_3$, $C_3$ = $C_1$/4) and Relu to $x_c$ were used to produce the result $x'_c$(1 x 1 x $C_3$). Then $x'_c$ continuously executed fully-convolution operation (channels: C1) and sigmoid activation, obtaining $x''_c$ (1 $\times 1 \times C_1$). The channel-wise multiplication of the input x and the $x''_c$ yields the channel attention map $x_{channel}$ (H x W x $C_1$). After adding two attention maps, convolution (kernel: [3, 3], stride: [1, 1]), batch normalization, and Relu are sequentially connected to obtain the output of the attention block.

**Fig. 4:**
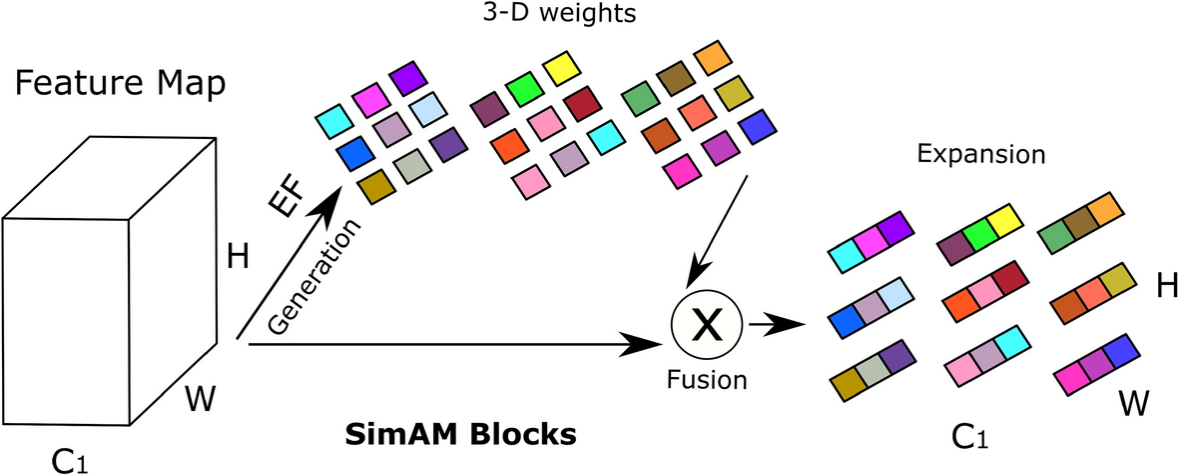
Structure of SimAM in Single information channel. H, C, and W represent height, channel, and width, respectively. Abbreviations: EF stands for energy function.

***SIC***: This channel can extract the best spatial information. The SimAM attention mechanism allocates weights to spatial dimension characteristics^134^. Fig 4 visually represents the architecture of the SimAM. The most relevant neurons in visual neuroscience exhibit different firing patterns in the surrounding neurons and maintain their activity, a phenomenon known as spatial suppression^135^. Measuring the linear separability between the target and other neurons is the quickest technique to identify these spatially suppressed neurons. The edge features of images frequently play a significant role in categorization problems in computer vision. In addition, spatial suppression neurons frequently display extraordinarily high contrast with the surrounding colors and textures, just like the edge elements of images. The energy function from neuroscience is thus used by the SimAM attention mechanism to assign weights to various spatial regions. The minimal energy of neurons can be represented in Eq. (1) because the energy function treats feature maps’ every pixel as an individual neuron.

1 $$\begin{aligned} { e^*_{x} } = \frac{4(\sigma ^2 + \omega )}{(x-\mu )^2 + 2\sigma ^2 + 2\omega } \end{aligned}$$

Where x is the target neuron, $\sigma$ and $\mu$ are variance and mean calculated over all neurons except the target neuron, and $\omega$ is the coefficient added to the variance to smoothen the variance effect, thereby controlling the attention mechanism’s sensitivity to the features’ variance. The coefficient $\omega$ is set to 1e - 4 as was used in CIFAR datasets by^134^. Spatial suppression neurons have a higher linear separability than other neurons, which results in a considerable *x* and $\mu$ deviation and a low $e^*_{x}$. In contrast, it is believed in neuroscience that neurons with lower energy are more distinct from nearby neurons. Therefore, using $e^*_{x}$, it is possible to determine each neuron’s weight. A scaling operator in Eq. (2) is used to reach the optimization phase of the entire SimAM attention mechanism.

2 $$\begin{aligned} { \tilde{F} = sigmoid \biggl (\frac{1}{E} \biggl ) F} \end{aligned}$$

where $\tilde{F}$ and *F* are output and input feature maps, all $e^*_{x}$ are grouped in channel and spatial dimensions and represented as *E*. A sigmoid is added to limit excessively high E values. So, the sigmoid activation function determines each neuron’s confidence at each location. The output of the SimAM block is a feature map with the same dimensions as the input block. However, the feature values are altered based on attention weights to highlight significant regions and hide less significant ones. This improves the model in drawing conclusions and learning from the most pertinent features found in the data. The VGG-16 model^84^ was introduced by the Visual Geometry Group (VGG). Its novel contributions were to increase network depths from 8 to 16 and split up large convolution kernels like 9 x 9 and 7 x 7 into multiple 3 x 3 small convolution kernels. Due to its deep and consistent architecture, which uses many layers of 3x3 convolutional filters, VGG16 excels at extracting spatial information. This design allows The network to record complex spatial patterns and hierarchies. VGG16 builds a hierarchy of feature maps with progressively decreasing spatial dimensions and increasing feature channels to encode low-level and high-level spatial details. Additionally, the pre-trained models of VGG16, which were trained on expansive datasets like ImageNet, offer a solid foundation for spatial feature extraction, making it an excellent option for computer vision tasks demanding accurate spatial understanding. After AlexNet^136^, it represents another major step in DL and serves as a benchmark for evaluating new approaches. The VGG model has a lot of benefits^137^. It uses a tiny convolution kernel to improve the extraction of spatial information.

**Fig. 5:**
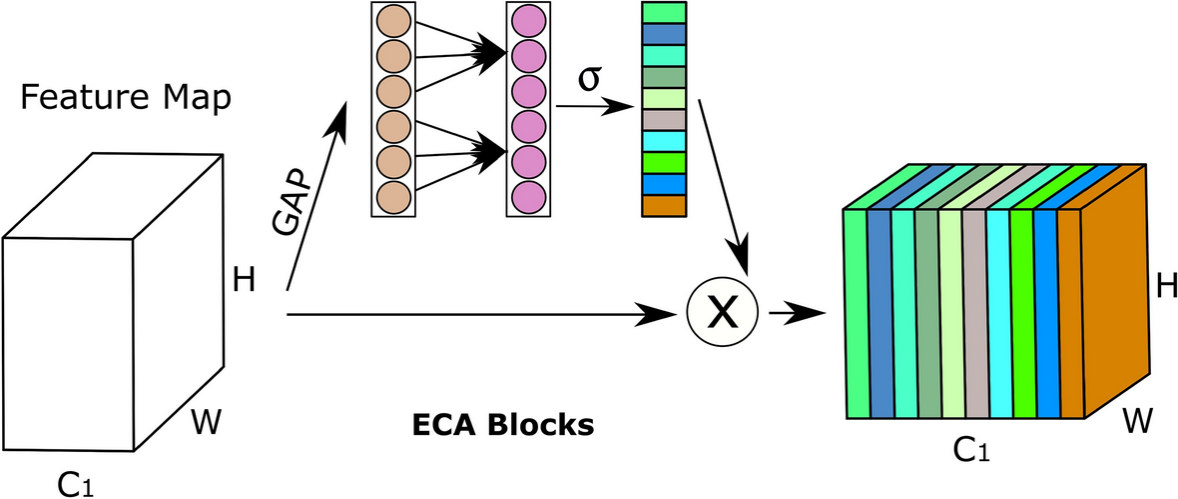
Structure of ECA module in Multi-scale spatial information channel. H, C, and W represent height, channel, and width, respectively. Abbreviations: GAP stands for global average pooling.

***MSIC:*** Depth-separable convolution of the Xception model^86^ is used to implement the MSIC channel. To properly extract multi-scale spatial information, depth-separable convolution diversifies the information derived from individual channels within the feature map. MSIC diversifies information extraction within each channel while efficiently capturing multi-scale spatial details. After each flow, the Xception model employs the ECA attention mechanism to improve its capacity to obtain data on multiple scales. The ECA attention mechanism uses a quick method to weigh the significance of each feature map’s channel information^46^. GAP is initially used by the ECA attention mechanism to collect channel-specific data, followed by 1D convolutional, which uses a convolutional kernel of size k to gather cross-channel interaction data, and finally, the sigmoid activation function to gather channel-wide weight data. This innovative approach enhances the model’s ability to extract valuable features and optimizes computational resources, making it an excellent choice for tasks requiring precise multi-channel attention. Fig. 5 presents the ECA attention mechanism architecture. The depth-separable convolution and residual mechanism are combined in the Xception model^86^ to enhance the Inception-V3 model^87^. Contrary to conventional convolution, depth-separable convolution carries out each channel in the feature map independently^138^. The benefit of Xception is the integration of depth-separable convolution with residual structure. The image’s multi-scale characteristics are successfully extracted using depth-separable convolution, and the network model converges quickly thanks to the residual method. The tiny convolutional kernel in depth-separable convolution offers the Xception model excellent local multi-scale information extraction capabilities, in contrast to the Inception-V3 model.

***Multi-channel ensemble strategy:*** By combining the strength of numerous data sources or channels, a multi-channel fusion strategy can greatly improve classification performance. This methodology can generate complementary insights by combining data from many channels, increasing the feature representation, and enhancing the model’s ability to distinguish across classes. To increase classification performance, this method uses an integrated classifier that depends on the weights and classification decision values of various channels^69^. Additionally, it encourages robustness by allowing the classifier to adjust to changes and difficulties in particular channels, lessening the influence of noise or uncertainties. It optimizes decision-making processes through sophisticated fusion techniques, such as weighted voting or feature concatenation, reducing the likelihood of misclassifications and bolstering overall accuracy. This method essentially combines many data streams into a single, comprehensive perspective, producing a categorization system that is more accurate and efficient across a variety of applications and domains. In this experiment, classification decision values for each channel utilizing pooling, fully connected, and softmax layers are obtained using the most recent feature maps of MGIC, SIC, and MSIC. To produce the classification decision values for the MCAM model, the classification decision values for each channel are then weighted and evaluated using grid-weighted voting. The formula for weighted majority voting combines the votes of multiple classifiers, each weighted by its importance or reliability. The final classification decision is that the class label receives the most weighted votes. Let $w_{i}$ be the weight and $v_{j}$ be the vote of a channel for label $l_{j}$. The weighted vote for class label $l_{j}$ is computed using Eq. (3).

3 $$\begin{aligned} { V(j) = \sum \limits _{i=1}^{n} \omega _{i} v_{i}(j)} \end{aligned}$$

The category included in the MCAM model’s maximum classification decision values is then used as the final classification outcome. The final classification decision *C* is the class label with the highest weighted vote, which is calculated using Eq. (4).

4 $$\begin{aligned} { C = argmax_{j\in {\{1, . . . , k\}}} V(j)} \end{aligned}$$

In short, the weighted vote for each class label is computed, followed by determining the class label with the highest weighted vote. This ensures the final decision considers individual classifiers’ votes and their respective weights, leading to a reliable classification.

The feature map *F* is defined as $F\in R^{C_{1}.H.W}$. All the input feature points $x_i$ share weights with the *M* input and $\hat{M}$ output channels. The feature map is fed into a convolutional layer $\{A,B,C\}\in R^{\hat{M}.H.W}$ are reshaped $\{A,B,C\}\in R^{\hat{M}.N}$, where N is the feature map size. The A,C results $\{A,C\}\in R^{\hat{M}.N}$ after transpose. A matrix multiplication of *A* and *B* performed on each row generates the attention map as expressed in Eq. (5).

5 $$\begin{aligned} AM_{ab} = \frac{e \sum \limits _{k=1}^{\hat{M}} A_{ak} B_{kb}}{\sum \limits _{\omega =1}^{N} e \sum \limits _{k=1}^{\hat{M}} A_{ak} B_{kb}} \end{aligned}$$

The channel attention module’s input-output relation is expressed in the Eq. (6).

6 $$\begin{aligned} { z_{a} = \frac{1}{c(x)} \sum \limits _{\forall a} f(x_a, x_b) x_b} \end{aligned}$$

$x_a$ and $z_a$ are the channel’s input and output feature maps. To reduce the calculation, the feature map is expanded into 1-D column vectors, $\{x_i, x_j\} \in R^{N}$. The correlation function is defined in the Eq. (7).

7 $$\begin{aligned} { f(x_{a},x_{b}) = e^{Q((x_{a} - Q(x_{a})) . (x_{b} - Q(x_{b})))}} \end{aligned}$$

$Q((x_{a} - Q(x_{a})) . (x_{b} - Q(x_{b})))$ is the covariance of $x_{a}$ and $x_{b}$, $Q(x_a)$ is approximate by mean of $x_a .Q(x_a,x_b)$ as shown in the Eq. (8).

8 $$\begin{aligned} { f(x_{a},x_{b}) = e^\frac{{Q((x_{a} - Q(x_{a})) . (x_{b} - Q(x_{b})))}}{N}} \end{aligned}$$

The operational approach of the multi-channel ensemble model is outlined through Algorithm 1.

**Algorithm 1:**
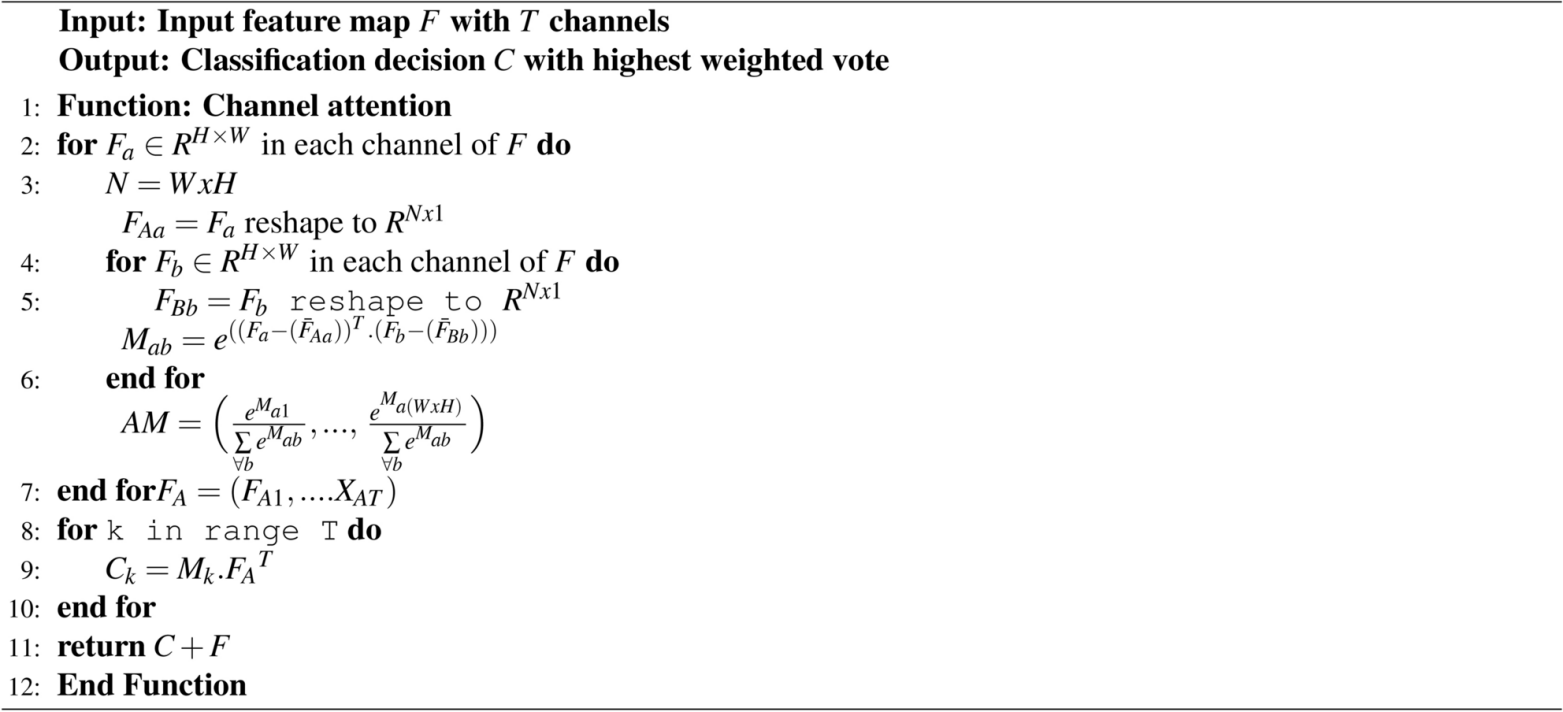
Pseudocode of the algorithm followed by each channel in the ensemble framework.

## Experimental results and analysis

This section delves into the experimental setup, giving an overview of the conditions that led to the thorough testing of our proposed framework. A detailed discussion of the classification experiment results and an analysis of the long-term experiment results are provided. This thorough investigation seeks to provide a nuanced understanding of our experimental setup’s performance metrics and results. Through thoroughly examining the results under various conditions and scenarios, we offer readers a thorough understanding of the efficiency and resilience of our suggested framework in various experimental settings, thus assisting in a comprehensive assessment of its capabilities.

### Experimental environment

This section thoroughly investigates the experimental environment, covering essential components like dataset information, dataset partitioning, experimental parameter configurations, and evaluation metrics used to gauge the effectiveness of the suggested framework. A thorough analysis of the segmentation procedures and comprehensive insights into the make-up and properties of the datasets used in our experiments are presented. Comprehensive explanations of the experimental parameter settings critical to the framework’s functionality provide insight into the decisions made during the experimentation process. Moreover, the assessment metrics employed to determine the efficacy of the suggested framework are elaborated upon, offering a thorough summary of the methodological factors and standards utilized for a comprehensive appraisal of its capacities.

#### Dataset

**Fig. 6:**
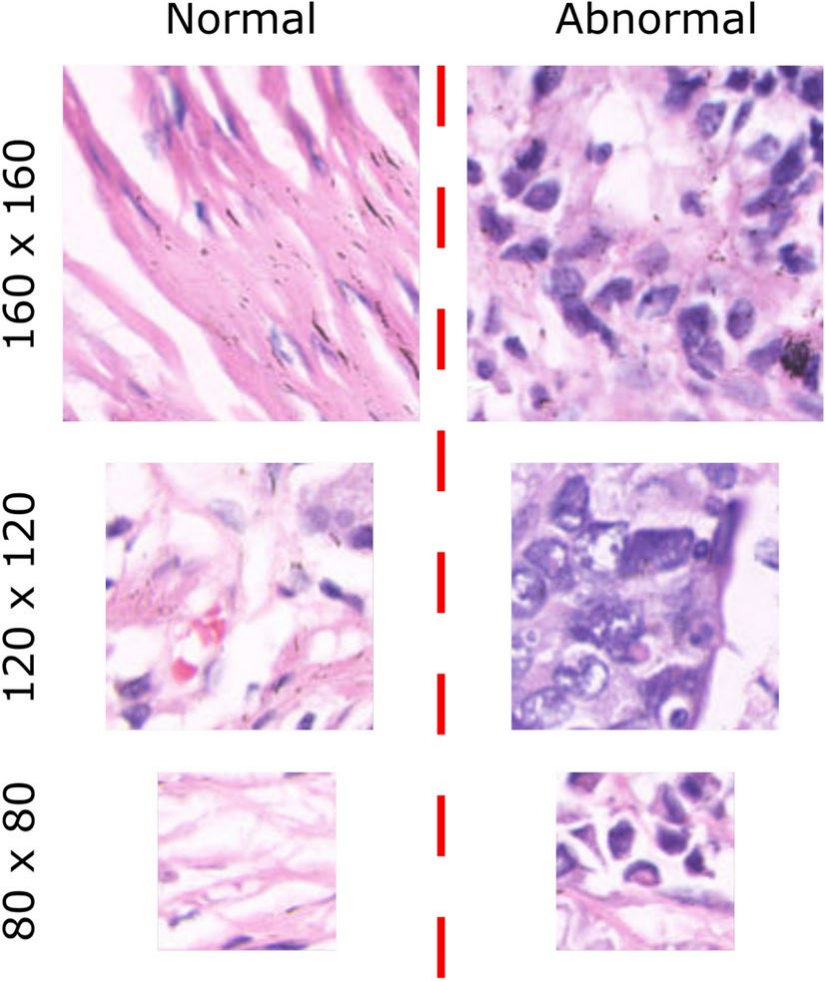
Sample images from the GasHisSDB Database: Sub-datasets A, B, and C with resolutions of 160x160, 120x120, and 80x80 Pixels, respectively, showcasing both normal and abnormal class samples.

GasHisSDB is a recently available histopathology image dataset with 245196 images. The dataset is divided into three sized cropped sub-size image datasets of 160x160, 120x120, and 80x80 pixels. Each sub-size dataset contains separate folders of normal and abnormal images. The total number of all normal and abnormal images is approximately 148120 and 97076, respectively. Table 1 shows the GasHisSDB dataset distribution. The normal images are generally free from any cancerous region. In addition, the nuclei of the cells in the micrograph are regularly arranged in a single layer with essentially little mitosis^67^. Therefore, it can be determined that an image under an optical microscope is normal if no cancellation of any cells or tissues is seen and the parameters of a normal image are met^139^. The abnormal images with malignant cells show that GC typically takes the form of an ulcer. Cancer nests spread as the condition worsens, invading the muscle, serosal, and mucosal layers. It has a rough texture and is frequently gray or white. The cancer cells can be grouped in a nest, acinar, tubular, or cord shape when observed under a microscope, and the border with the stroma is typically distinct. However, the line dividing the cancer cells from the stroma is blurred when they invade it^67^. Normal and abnormal sample images from three sub-size datasets, A, B, and C, are shown in Fig. 6. Based on the aforementioned information, it is possible to determine that the pathological image is aberrant when cells are seen to form gland or adenoid structures that are uneven in size, varied in shape, or arranged irregularly. The malignant cells are frequently irregularly distributed in multiple layers in the abnormal images, and the nuclei display a variety of sizes and division phenomena^15,140–142^. The GasHisSDB dataset contains diverse collection of histopathology images, captured under different imaging conditions and representing a wide range of patient cases. This dataset includes variations in staining techniques, and tissue structures, making it well-suited for evaluating the robustness of the proposed framework. Additionally, it consists of both normal and abnormal samples across multiple resolution levels (160$\times$160, 120$\times$120, and 80$\times$80 pixels), ensuring a comprehensive assessment of the model’s ability to adapt to different image scales. The structured nature of the dataset facilitates rigorous testing, allowing the model to learn discriminative features essential for accurate GC classification across varied clinical scenarios.

**Table 1:** GasHisSDB dataset distribution description.

Sub-dataset	Cropping size	Normal	Abnormal
Sub-dataset A	160 x 160	20,160	13,124
Sub-dataset B	120 x 120	40,460	24,801
Sub-dataset C	80 x 80	87,500	59,151
Total		148,120	97,076

#### Data setting

GasHisSDB’s dataset distribution technique has been developed for extensive evaluation and reliable model training. We used a meticulous technique for each sub-dataset A, B, and C separately. First, each sub-dataset, featuring both normal and abnormal classes, is subjected to a randomized split into training and testing sets, maintaining a proportional 70:30 distribution. Further segmentation of the training data is also performed, with the training and validation sets being randomly assigned at a 70:30 ratio. A refined model creation approach, including training and validation phases, is made possible by this internal division. Importantly, to ensure the randomization of data splits, the training and validation sets are assigned to the training data four times at random. This strategy reduces the effects of data partitioning randomness on the outcomes and ensures the robustness of the performance evaluation of the ML model. Moreover, the model’s generalization abilities can be better understood due to this data distribution and experimental approach, which also ensures the model’s performance is reliable and independent of any specific random partition.

The images were normalized using min-max normalization to scale the pixel intensity values to a standard range. This ensures uniformity in the data, reduces the effects of varying pixel intensities, and enhances the efficiency of the training process for deep learning models. The normalization was applied to each pixel intensity value *x* in the images using the Eq. (9).

9 $$\begin{aligned} x' = \frac{x - \min (x)}{\max (x) - \min (x)} \end{aligned}$$

The data settings for sub-datasets A, B, and C are listed in Table 2, 3, and 4, respectively.

**Table 2:** Sub-dataset A distribution for training, validation, and testing.

Image class	Training	Validation	Testing
Normal	10,342	4,722	5,096
Abnormal	5,967	2,268	4,889
Sum	16,309	6,990	9,985

**Table 3:** Sub-dataset B distribution for training, validation, and testing.

Image class	Training	Validation	Testing
Normal	20,972	7,882	11,606
Abnormal	11,006	5,823	7,972
Sum	31,978	13,705	19,578

**Table 4:** Sub-dataset C distribution for training, validation, and testing.

Image class	Training	Validation	Testing
Normal	47,340	13,313	26,847
Abnormal	24,519	17,484	17,148
Sum	71,859	30,797	43,995

**Table 5:** Complete GasHisSDB database distribution for training, validation, and testing.

Image class	Training	Validation	Testing
Normal	75,264	25,494	47,362
Abnormal	44,882	25,997	26,197
Sum	120,146	51,491	73,559

#### Hyper-parameters setting

To achieve optimal performance on the GasHisSDB dataset, the hyperparameter settings for the proposed MCAM model were empirically tuned. Based on preliminary tests, the main hyperparameters, including learning rate, batch size, and optimizer settings were methodically changed to strike a balance between generalization, stability, and training effectiveness. The model was trained for 100 epochs with a batch size of 16 chosen after experimenting with smaller and larger values, where smaller batch sizes led to increased gradient noise, and larger batch sizes resulted in higher memory requirements without significant performance raise. The learning rate was set to $2 \times 10^{-3}$, selected after testing a range of values between $1 \times 10^{-4}$, and $1 \times 10^{-2}$. The chosen value provided a suitable balance between convergence speed and stability, ensuring effective optimization without overshooting the minima. The AdamW stochastic optimizer was used for optimization due to its ability to effectively handle weight decay, which is critical for regularization. The optimizer’s parameters were carefully configured as follows: the epsilon $(\epsilon )$ was set to $1 \times 10^{-8}$ to ensure numerical stability during gradient updates, the weight decay was set to $1 \times 10^{-2}$ to regularize the model and prevent overfitting, and the momentum parameters $(\beta _{1}, \beta _{2})$ were set to [0.9, 0.999], which are commonly used defaults for Adam-based optimizers and provide a balance between convergence speed and model generalization.

The model parameters were assessed on the validation set following each training cycle to guarantee strong generalization. The training process was conducted with the parameters that yielded the best validation accuracy. With this method, it was guaranteed that the model configuration with the best performance would be used for additional testing and assessment. Furthermore, early stopping was used to end training if no discernible improvement was seen on the validation set over a predetermined number of consecutive epochs, even though the model was trained for a maximum of 100 epochs. This helped to mitigate overfitting. These steps, combined with the modified transfer learning approach discussed above, provided a systematic framework for parameter tuning and optimization, ensuring that the MCAM model achieved high accuracy and robustness for GC classification.

#### Evaluation metrics

Selecting the right evaluation criteria is essential to overcoming bias between different algorithms. The most common measures for assessing classification performance are sensitivity (Sens.), specificity (Spec.), average accuracy (Avg. Acc.), and F1-score. The above-mentioned metrics are defined by using True positive (TP), False positive (FP), True negative (TN), and False negative (FN). The assessment parameters Sesn., Spec., Avg. Acc., F1, Pre., and Rec. are calculated using Eqs. (10), (11), (12), (13), and (14), respectively. Sensitivity, also known as recall, measures the proportion of positively classified samples to all positively classified samples. Contrarily, specificity measures the model’s capacity to distinguish negative instances accurately and represents the proportion of real negatives to all other negatives. A key indicator of how well a model predicts outcomes is accuracy, which considers both true positives and negatives concerning all samples. Accuracy is the most typical and fundamental evaluation criterion. When aiming for a unified evaluation of classification models, the F1 score’s combination of precision and recall provides a thorough review that balances the trade-off between false positives and false negatives. Precision calculates the proportion of TP results among all positive predictions made by the model. In contrast, Recall calculates the proportion of TP among all actual positive instances. These metrics help evaluate and improve a model’s performance for particular application domains by providing critical insights into a model’s strengths and flaws.

The evaluation metrics used in this study are clinically significant in GC classification. Sensitivity is crucial to ensure that positive cases are correctly identified, thereby reducing the likelihood of missed cancer diagnoses, which can lead to delayed treatment. High specificity is equally important, as it minimizes false positives, preventing unnecessary invasive procedures such as biopsies. The F1-score, which balances precision and recall, is particularly useful in histopathology image classification, where an imbalance between normal and abnormal samples can impact model reliability. A high F1-score indicates that the model performs well across both categories, ensuring a more dependable decision-support tool for pathologists. By achieving high values in these metrics, our proposed framework demonstrates its potential for clinical application, aiding in accurate, efficient, and early detection of GC.

10 $$\begin{aligned} Sens. = \frac{TP}{TP+FN} \end{aligned}$$

11 $$\begin{aligned} Spec. = \frac{TN}{TN+FP} \end{aligned}$$

12 $$\begin{aligned} Avg. Acc. = \frac{TP+TN}{TP+TN+FP+FN} \end{aligned}$$

13 $$\begin{aligned} F1 = \frac{2 \times TP}{2 \times TP+FP+FN} \end{aligned}$$

14 $$\begin{aligned} Pre. = \frac{TP}{TP+FP} \end{aligned}$$

**Fig. 7:**
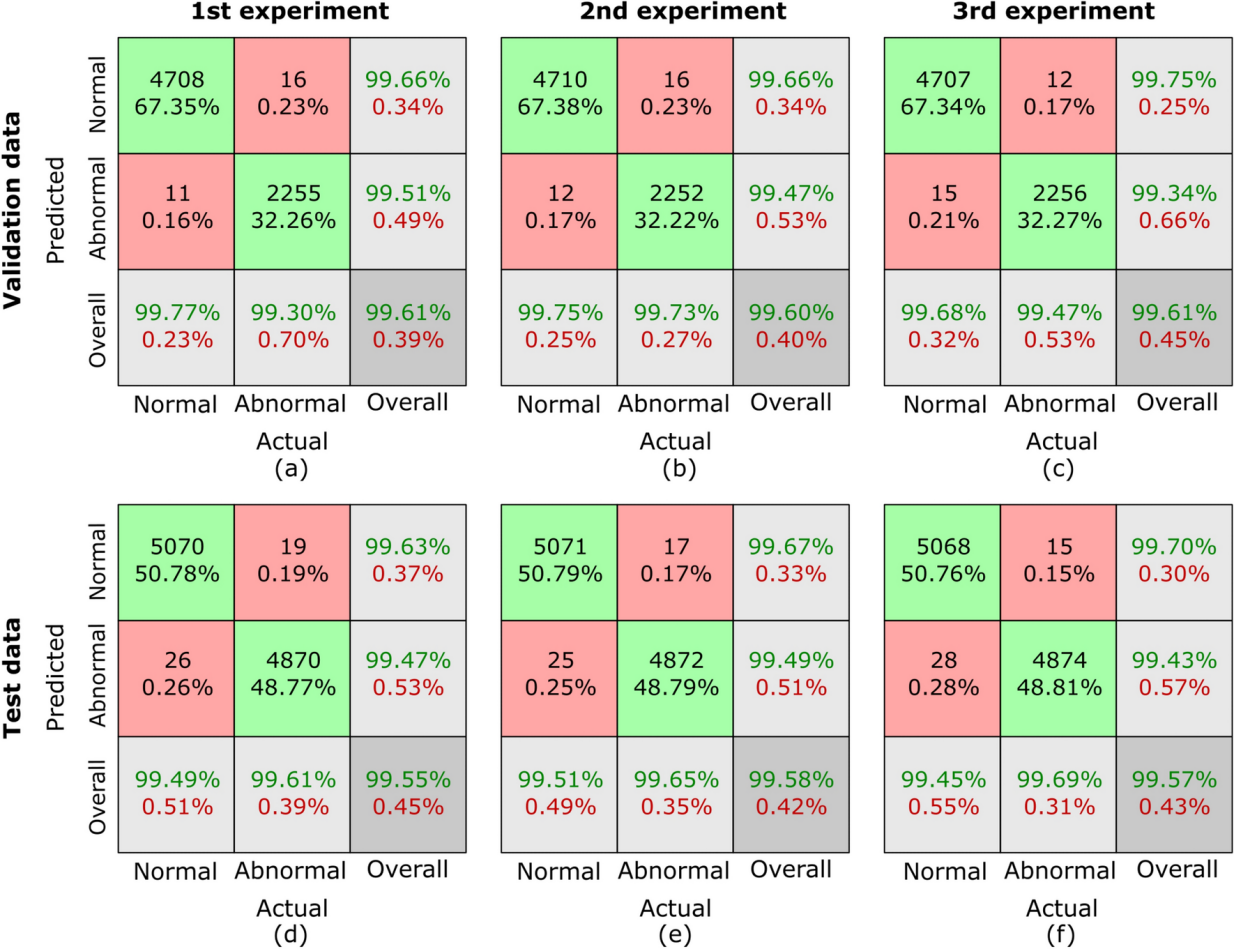
Confusion matrices for sub-dataset A from three randomized experiments using the proposed MCAM model. (**a**)-(**c**) represent results on validation data, while (**d**)-(**f**) correspond to results from randomized experiments on the testing dataset. Each column corresponds to one experiment. The green blocks indicate the counts and percentages of true positive and true negative cases, while the red blocks represent false positive and false negative cases. In the last row, the first block shows sensitivity for normal cases and specificity for abnormal cases, the middle block shows sensitivity for abnormal cases and specificity for normal cases, and the last block represents the overall classification accuracy as a percentage. This visualization highlights the model’s consistent performance across all experiments.

**Fig. 8:**
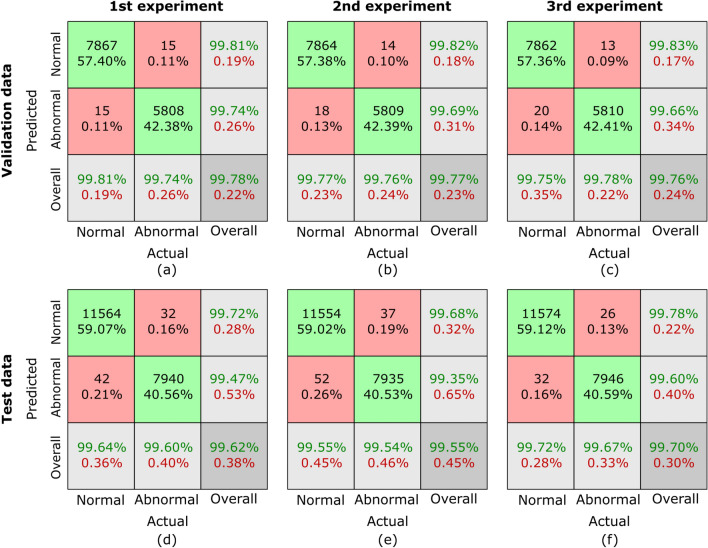
Confusion matrices for sub-dataset B from three randomized experiments using the proposed MCAM model. (a)-(c) represent results on validation data, while (d)-(f) correspond to results from randomized experiments on the testing dataset. Each column corresponds to one experiment. The green blocks indicate the counts and percentages of true positive and true negative cases, while the red blocks represent false positive and false negative cases. In the last row, the first block shows sensitivity for normal cases and specificity for abnormal cases, the middle block shows sensitivity for abnormal cases and specificity for normal cases, and the last block represents the overall classification accuracy as a percentage. This visualization highlights the model’s consistent performance across all experiments.

**Fig. 9:**
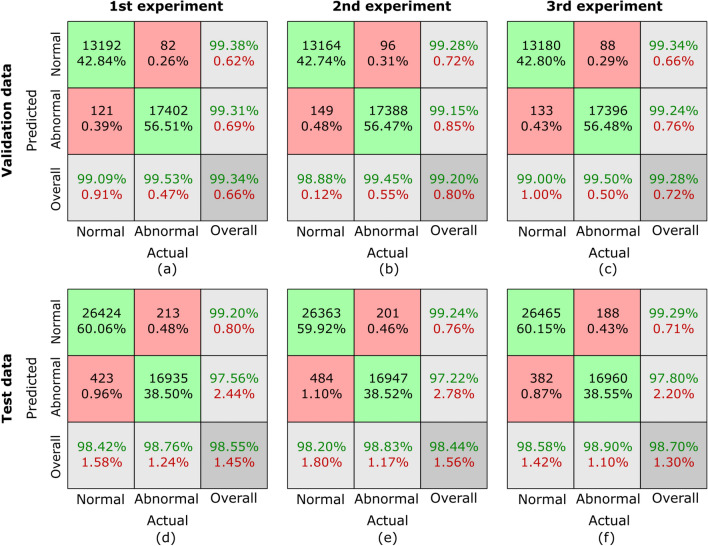
Confusion matrices for sub-dataset C from three randomized experiments using the proposed MCAM model. (**a**)-(**c**) represent results on validation data, while (**d**)-(**f**) correspond to results from randomized experiments on the testing dataset. Each column corresponds to one experiment. The green blocks indicate the counts and percentages of true positive and true negative cases, while the red blocks represent false positive and false negative cases. In the last row, the first block shows sensitivity for normal cases and specificity for abnormal cases, the middle block shows sensitivity for abnormal cases and specificity for normal cases, and the last block represents the overall classification accuracy as a percentage. This visualization highlights the model’s consistent performance across all experiments.

**Fig. 10:**
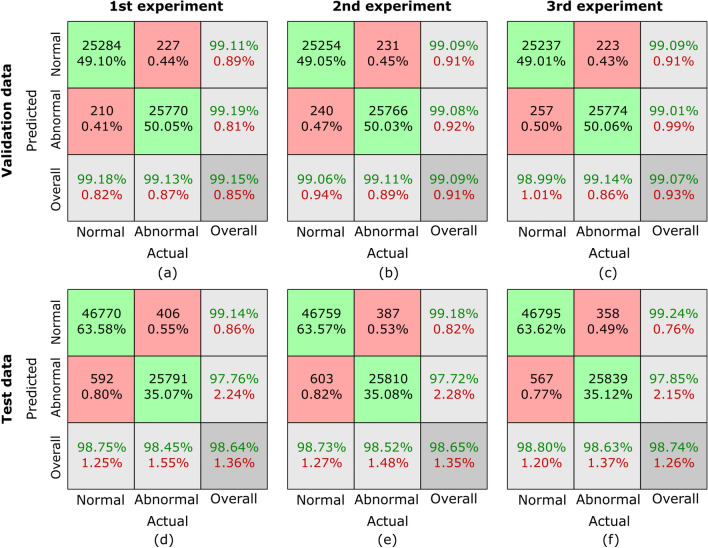
Confusion matrices for complete GasHisSDB database from three randomized experiments using the proposed MCAM model. (**a**)-(**c**) represent results on validation data, while (**d**)-(**f**) correspond to results from randomized experiments on the testing dataset. Each column corresponds to one experiment. The green blocks indicate the counts and percentages of true positive and true negative cases, while the red blocks represent false positive and false negative cases. In the last row, the first block shows sensitivity for normal cases and specificity for abnormal cases, the middle block shows sensitivity for abnormal cases and specificity for normal cases, and the last block represents the overall classification accuracy as a percentage. This visualization highlights the model’s consistent performance across all experiments.

### Classification assessment

This section provides a detailed analysis of the performance of our suggested model by presenting a thorough exposition of the experimental results obtained on both sub-datasets and the entire dataset. The conversation includes in-depth analyses of contrast experiment results, illuminating how our model compares to pertinent industry standards. Furthermore, we perform extensive experiments to thoroughly evaluate the adaptability and stability of our suggested model in a range of scenarios. This comprehensive analysis provides a nuanced understanding of the model’s performance across various data subsets and its flexibility to different experimental scenarios, thereby aiding in a comprehensive assessment of its effectiveness and potential utility.

#### Experimental results

Confusion matrices are generated to comprehensively assess the outcomes of our proposed MCAM model on three randomized experiments on sub-dataset A, B, C, and the whole database GasHisSDB. A confusion matrix is invaluable for analyzing a model’s performance in a classification challenge. It offers a succinct description of how closely the model’s predictions match the labels from the actual ground truth (GT). Figure 7, 8, 9, and 10 represents the confusion matrices for three randomized experiments of sub-datasets A, B, C, and whole GasHisSDB database, respectively.

In the presented confusion matrices, the 1st column provides a detailed breakdown of results for the normal class. True negative (TN) instances are indicated by the 1st-row values, which are given as a percentage of TNs to all input samples. False positive (FP) cases are indicated in the second row by the percentage of FPs in all input samples. The last row displays the normal cases’ percentage sensitivity (in green). The first row in the 2nd column shows false negatives (FN) and their percentage relative to the total number of samples. True positives (TP) are displayed in the second row, along with their percentage value from all the input data samples. The final row displays the normal cases’ specificity percentage (in green text). In the 3rd column, the 1st row highlights the percentage value (in green text) of TN cases from the sum of TNs’ and FNs’ cases. The 2nd row displays the percentage value of the FP cases (in green text) from the sum of FPs and TPs. The last row provides the overall accuracy percentage value in green text. This detailed breakdown offers a comprehensive view of the performance metrics associated with each category. Moreover, the values of these randomized experiments with the average values are mentioned in Table 6. The highest average values are highlighted in bold to facilitate reader comprehension, while the second highest values are underlined. Table 6 provides a thorough performance evaluation of the proposed model, showing its efficacy using the evaluation metrics, including sensitivity, specificity, average accuracy, and the F1-score for sub-datasets A, B, and C, and the whole GasHisSDB dataset in separate.

In Table 6, it is evident that the average accuracy of the proposed MCAM model surpasses 99.50% for sub-datasets A and B. However, for sub-dataset C, the average accuracy declines to 98.31%. This discrepancy can be attributed to the lower resolution of the images in sub-dataset C, which inherently provides fewer detailed features for the model to analyze than higher-resolution images. Consequently, the model’s classification performance is slightly compromised on this subset. To address this concern comprehensively, we will analyze these results in the context of the samples and provide detailed explanations for each comparison, highlighting the best values using bold font to ensure clarity and emphasis. The detailed experimental findings for all sub-datasets and the complete dataset will be elaborated upon in the subsequent section, facilitating a comprehensive understanding of the model’s performance across different scenarios.

**Sub-dataset A:** In the 1st experiment, for the validation set, see Fig. 7 (a), 11 images in the normal category were incorrectly identified as abnormal. In comparison, 16 abnormal images were incorrectly classified as normal. For the test set, in Fig. 7 (d), 26 normal images were incorrectly identified as abnormal, whereas 19 abnormal images were incorrectly classed as normal.

In the 2nd experiment, for the validation set, Fig. 7 (b) shows 12 images in the normal category were mistakenly labeled as abnormal, whereas 16 abnormal images were wrongly labeled as normal. In the test set, Fig. 7 (e) shows 25 normal images were wrongly classified as abnormal, whereas 17 abnormal images were incorrectly classified as normal.

In the third experiment, for the validation set, see Fig. 7 (c), 15 images in the normal category were incorrectly identified as abnormal, and 12 abnormal images were incorrectly classified as normal. However, for the test set, 28 normal images were incorrectly identified as abnormal, whereas 15 abnormal images were incorrectly classed as normal see Fig. 7 (f).

For the sub-dataset A, the sensitivity, specific, F1-score, and precision values of all three randomized experiments are 99.71/99.40, 99.40/99.71, 99.58/99.56, 99.36/99.46 for Normal/Abnormal classes, on the validation set, respectively. However, these values are calculated for the testing set as 99.48/99.48, 99.98/99.48, 99.57/99.57, and 99.66/99.43. The average accuracy on validation and testing sets are 99.94 and 99.57, respectively.

**Sub-dataset B:** In the 1st experiment, for the validation set, see Fig. 8 (a), 15 images in the normal category were incorrectly identified as abnormal, and the same numbers of abnormal images were incorrectly classified as normal. For the test set, in Fig. 8 (d), 42 normal images were incorrectly identified as abnormal, whereas 32 abnormal images were incorrectly classed as normal.

In the 2nd experiment, for the validation set, Fig. 8 (b) shows 18 images in the normal category were mistakenly labeled as abnormal, whereas 14 abnormal images were wrongly labeled as normal. In the test set, Fig. 8 (e) shows 52 normal images were wrongly classified as abnormal, whereas 37 abnormal images were incorrectly classified as normal.

In the third experiment, for the validation set, see Fig. 8 (c), 20 images in the normal category were incorrectly identified as abnormal, and 13 abnormal images were incorrectly classified as normal. However, 32 normal images were incorrectly identified as abnormal for the test set, whereas 26 abnormal images were incorrectly classed as normal see Fig. 8 (f).

The evaluation metrics are presented in Table 6 for the sub-dataset B. For the validation set, sensitivity, specificity, and F1-score values of all three randomized experiments are 99.61/99.76, 99.76/99.61, and 99.69/99.68 for normal/abnormal classes, respectively. However, these values are computed for the testing set as 99.97/99.94, 99.94/99.97, and 99.95/99.95. The average accuracy on validation and testing sets are 99.94 and 99.60, respectively.

**Sub-dataset C:** In the 1st experiment, for the validation set, see Fig. 9 (a), 121 images in the normal category were incorrectly identified as abnormal, and 82 abnormal images were incorrectly classified as normal. For the test set, in Fig. 9 (d), 423 normal images were incorrectly identified as abnormal, whereas 213 abnormal images were incorrectly classed as normal.

In the 2nd experiment, for the validation set, Fig. 9 (b) shows 149 images in the normal category were mistakenly labeled as abnormal, whereas 96 abnormal images were wrongly labeled as normal. In the test set, Fig. 9 (e) shows 484 normal images were wrongly classified as abnormal, whereas 201 abnormal images were incorrectly classified as normal.

In the third experiment, for the validation set, see Fig. 9 (c), 133 images in the normal category were incorrectly identified as abnormal, and 88 abnormal images were incorrectly classified as normal. However, for the test set, 382 normal images were incorrectly identified as abnormal, whereas 188 abnormal images were incorrectly classed as normal see Fig. 9 (f).

The evaluation metrics are presented in Table 6 for the sub-dataset C. For the validation set, all three randomized experiments’ sensitivity, specificity, and F1-score values are calculated as 99.08/99.13, 99.13/99.08, and 99.25/99.24 for normal/abnormal classes, respectively. However, these values are computed for the testing set as 98.40/99.16, 99.16/98.40, and 98.62/98.95. The average accuracy on validation and testing sets are 99.48 and 98.31, respectively.

A comprehensive error analysis was conducted to examine misclassification patterns, with a specific focus on false positives and false negatives. The primary reason for misclassifications was the reduced resolution of 80$\times$80 pixel images, which resulted in a loss of structural details crucial for distinguishing between normal and abnormal tissue. Additionally, some cancerous and non-cancerous regions exhibited overlapping morphological features, leading to occasional confusion in classification. False positives were observed in cases where normal tissue contained irregular structural formations, causing the model to misclassify them as abnormal. Conversely, false negatives occurred in cases where cancerous regions had mild morphological variations, making them appear similar to normal tissues.

**Complete GasHisSDB database:** In the 1st experiment, for the validation set, see Fig. 10 (a), 210 images in the normal category were incorrectly identified as abnormal, and 227 abnormal images were incorrectly classified as normal. For the test set, in Fig. 10 (d), 592 normal images were incorrectly identified as abnormal, whereas 406 abnormal images were incorrectly classed as normal.

In the 2nd experiment, for the validation set, Fig. 10 (b) shows 240 images in the normal category were mistakenly labeled as abnormal, whereas 231 abnormal images were wrongly labeled as normal. In the test set, Fig. 10 (e) shows 603 normal images were wrongly classified as abnormal, whereas 387 abnormal images were incorrectly classified as normal.

In the third experiment, for the validation set, see Fig. 10 (c), 257 images in the normal category were incorrectly identified as abnormal, and 223 abnormal images were incorrectly classified as normal. However, for the test set, 567 normal images were incorrectly identified as abnormal, whereas 358 abnormal images were incorrectly classed as normal see Fig. 10 (f).

The evaluation metrics for the GasHisSDB as a whole dataset are presented in Table 6. For the validation set, all three randomized experiments’ sensitivity, specificity, and F1-score values are calculated as 99.08/99.13, 99.13/99.08, and 99.10/99.10 for normal/abnormal classes, respectively. However, these values are computed for the testing set as 98.76/98.53, 98.53/98.76, and 98.98/98.98. The average accuracy on validation and testing sets are 99.07 and 98.48, respectively.

**Fig. 11:**
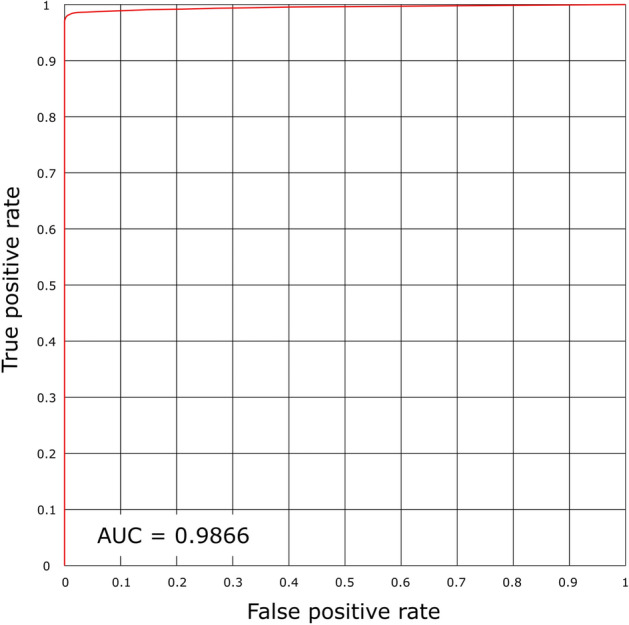
Graph of Sens to 1-Spec obtained using the MCAM model for classification.

Figure 11 shows the graph of Sen according to the value of 1 - Spec obtained using the MCAM model for classification. If the curve is close to the upper-left corner, it shows a large value of area under the curve (AUC) and high accuracy. As shown in Fig. 15, the model achieves AUC value of 0.9866.

An exciting finding from examining the model’s performance is that the differences between the test and validation sets’ accuracies remain remarkably small, never exceeding 1.00%. This result highlights the excellent extensibility and resilience of the proposed MCAM model, which is an integral characteristic. The model’s stability and capacity for effective generalization are highlighted because it maintains constant accuracy levels across various datasets during training and validation and when exposed to fresh, untried data (the test set). Such minor differences in accuracy between validation and test sets show the model’s ability to adjust to different data distributions and support its potential as a reliable tool.

**Table 6:** Performance evaluation of the proposed MCAM model in three randomized experiments on validation and testing sets. The best-achieved results for all sub-datasets are in bold, whereas the second-best results are underlined. “C” is the class that has “N” and “A,” which represent normal and abnormal labels, whereas GHS represents the complete GasHisSDB dataset. [Values in %].

Set	Exp	Class	Validation					Testing				
			Sens	Spec	F1	Pre	Acc	Sens	Spec	F1	Pre	Acc
A	1st	N	99.77	99.30	99.50	99.30		99.49	99.61	99.55	99.60	
		A	99.30	99.77	99.61	99.50	99.61	99.61	99.49	99.56	99.40	99.55
	2nd	N	99.75	99.73	99.51	99.30		99.65	99.58	99.70	99.62	
		A	99.73	99.75	99.52	99.50	99.60	99.65	99.51	99.58	99.50	**99.58**
	3rd	N	99.68	99.47	99.58	99.50		99.45	99.69	99.57	99.70	
		A	99.47	99.68	99.58	99.40	99.61	99.69	99.45	99.57	99.40	99.57
	Avg	N	99.71	99.40	99.58	99.36		99.48	99.98	99.57	99.66	
		A	99.40	99.71	99.56	99.46	99.94	99.98	99.48	99.57	99.43	99.57
B	1st	N	99.81	99.74	99.77	99.70		99.64	99.60	99.62	99.60	
		A	99.74	99.81	99.78	99.70	99.78	99.60	99.64	99.62	99.40	99.62
	2nd	N	99.77	99.76	99.52	99.76		99.55	99.54	99.55	99.40	
		A	99.76	99.77	99.51	99.69	99.77	99.54	99.55	99.55	99.30	99.55
	3rd	N	99.75	99.78	99.77	99.78		99.72	99.67	99.69	99.70	
		A	99.78	99.75	99.76	99.66	99.76	99.67	99.72	99.69	99.60	**99.70**
	Avg	N	99.61	99.76	99.69	99.75		99.97	99.94	99.95	99.57	
		A	99.76	99.61	99.68	99.68	99.94	99.94	99.97	99.95	99.43	99.60
C	1st	N	99.09	99.53	99.32	99.50		98.42	98.76	98.59	98.80	
		A	99.53	99.09	99.31	99.30	99.34	99.76	99.42	99.59	97.60	98.55
	2nd	N	98.88	99.45	99.17	99.50		98.20	98.83	98.52	98.80	
		A	99.45	98.88	99.16	99.20	99.20	98.83	98.20	98.51	97.20	98.44
	3rd	N	99.00	99.50	99.25	99.50		98.58	98.90	98.74	99.00	
		A	99.50	99.00	99.25	99.20	99.28	98.90	98.58	98.74	97.80	**98.70**
	Avg	N	99.08	99.13	99.25	99.50		98.40	99.16	98.62	98.86	
		A	99.13	99.08	99.24	99.23	99.48	99.16	98.40	98.95	97.53	98.31
GHS	1st	N	99.18	99.13	99.16	99.10		98.75	98.45	98.60	98.40	
		A	99.13	99.18	99.16	99.30	99.15	98.45	98.75	98.60	97.80	98.64
	2nd	N	99.06	99.11	99.08	99.10		98.73	98.52	98.63	98.50	
		A	99.11	99.06	99.08	99.20	99.09	98.52	98.73	98.63	97.80	98.65
	3rd	N	98.99	99.14	99.06	99.20		98.80	98.63	98.72	98.60	
		A	99.14	98.99	99.06	99.00	99.07	98.63	98.80	98.71	98.00	**98.74**
	Avg	N	99.08	99.13	99.10	99.13		98.76	98.53	98.72	98.50	
		A	99.13	99.08	99.10	99.16	99.07	98.53	98.76	98.50	97.87	98.48

subsubsection*Contrast experiments of GC diagnosis and classification The following are the three contrast investigations: The initial comparison assesses the MCAM framework against standard DL models, while the second scrutinizes its performance in contrast to models without TL. The third comparison evaluates the MCAM framework against models lacking attention mechanisms.

**Proposed MCAM versus competitive deep learning models:** To affirm the superior performance of our MCAM model framework in the task of GC diagnosis, we benchmark it against 18 different DL models, including ViT, CNN and MLP models. The VT models includes ViT^92^, CaiT^93^, DeiT^94^, CoaT^96^, BoTNet-50^98^, LeViT^97^, and T2T-ViT^95^. The CNN models are VGG-16^84^, Xception^86^, Inception-V3^87^, AlexNet^136^, DenseNet-121^85^, InceptionResNet-V1^89^, and ResNet-50^83^,and ResNeXt-50^88^. The MLP models, including gMLP^143^, MLP-Mixer^144^, and ResMLP^145^. A comparison of the DL models with our proposed MCAM model is shown in Table 7. The results obtained from the comparison analysis between the proposed MCAM framework and other DL models are reported in Table 7. The assessment of the proposed model’s performance measures involves aggregating outcomes from three randomized experiments performed on the complete GasHisSDB dataset. Within the normal category, EfficientNetV2 displayed the highest sensitivity levels 98.37% and F1-score 98.40%, while VGG-16 demonstrated the best specificity (98.50%). Conversely, in the abnormal group, Xception achieved the maximum sensitivity 98.55%, while Inception-V3 and provided the top values for specificity 98.71% and F1-score (98.24%). Notably, EfficientNetV2 displayed the highest average accuracy at 98.06%. The CNN models consistently outperformed other DL models. The suggested MCAM framework displayed higher performance than traditional DL models. MCAM’s assessment metrics with the best results from other traditional models demonstrated gains of 0.08, 0.63, and 0.75 for sensitivity, specificity, and F1-score, respectively, for the abnormal category. For the normal category, these values are calculated as 0.63, 0.03, and 0.72. Although these improvements may not be extremely high, they underline the suggested framework’s prospective characteristics.

The findings of the comparative experiment, which contrasted the performance of the suggested MCAM framework with that of classic DL approaches, demonstrate a remarkable advancement in the capabilities of the MCAM model for the task of GC detection and classification. The MCAM model greatly surpassed the traditional DL model in accuracy and effectiveness, emphasizing its more significant potential and efficacy in this crucial diagnostic task.

**Table 7:** Assessing the efficacy of the proposed model against conventional DL models using the test dataset. The best-achieved values are in bold, while the second-highest values are underlined. The top values are individually highlighted in bold and underlined for both normal and abnormal categories. “N” and “A,” represent normal and abnormal labels. [Values in %].

	Model	Class	Sens	Spec	F1	Pre	Avg. Acc
**VT**	ViT^92^	N	74.08	78.90	75.82	74.85	
		A	74.23	77.90	75.89	75.01	77.86
	CaiT^93^	N	75.68	72.44	75.74	73.25	
		A	73.44	75.68	73.73	73.31	74.80
	DeiT^94^	N	93.22	94.12	93.85	93.88	
		A	92.36	94.77	93.68	93.46	93.87
	CoaT^96^	N	73.89	81.25	80.25	80.22	
		A	87.58	73.89	82.32	83.14	80.92
	BoTNet-50^98^	N	95.24	94.28	95.20	95.40	
		A	93.25	94.52	95.65	93.82	95.03
	LeViT^97^	N	79.62	80.23	81.24	80.28	
		A	81.25	79.25	80.41	80.40	80.74
	T2T-ViT^95^	N	90.14	92.35	91.02	88.98	
		A	89.35	91.25	92.23	90.20	92.01
**CNN**	VGG-16^84^	N	96.72	98.50	97.72	95.68	
		A	98.45	96.72	97.69	95.66	97.72
	MCLNet^146^	N	97.57	97.03	97.77	97.78	
		A	97.03	97.57	96.67	95.66	97.36
	Xception^86^	N	97.85	98.47	98.23	98.32	
		A	**98.55**	98.71	98.24	97.42	97.98
	Inception-V3^87^	N	98.13	98.38	98.26	97.66	
		A	98.34	98.13	98.23	97.66	98.01
	AlexNet^136^	N	96.42	95.32	94.21	92.36	
		A	92.57	96.74	94.99	94.44	94.90
	DenseNet-121^85^	N	97.12	97.76	95.09	95.22	
		A	97.67	96.16	96.90	95.36	96.92
	MobileNetV3^56^	N	96.57	92.83	93.87	92.66	
		A	92.83	96.57	90.80	90.88	92.64
	InceptionResNet-V1^89^	N	95.47	96.74	96.40	95.78	
		A	95.55	95.23	95.32	94.22	96.12
	DenseNet-121^56^	N	96.57	92.83	93.87	94.82	
		A	92.83	96.57	90.80	89.66	92.64
	ResNet-50^83^	N	93.41	93.22	96.55	92.64	
		A	95.77	93.22	94.62	94.44	94.67
	EfficientNetV2^147^	N	98.37	97.60	98.40	98.42	
		A	97.60	98.37	97.53	97.12	98.06
**MLP**	gMLP^143^	N	89.23	88.32	89.60	87.56	
		A	88.69	88.70	88.24	87.66	88.51
	MLP-Mixer^144^	N	72.11	73.01	73.32	72.22	
		A	72.41	74.21	72.02	73.58	73.24
	ResMLP^145^	N	72.12	78.41	75.21	74.22	
		A	73.21	77.14	75.21	73.24	74.73
**Proposed**	**MCAM**	N	**98.76**	**98.53**	**98.72**	**98.50**	
		A	98.53	**98.76**	**98.50**	**97.87**	**98.48**

**Proposed MCAM versus competitive ensemble models:** To evaluate the performance of the proposed MCAM model, it was compared against state-of-the-art hybrid and ensemble models from competitive studies. As shown in Table 8, our proposed model consistently outperforms existing models across all sub-datasets. Although previously reported accuracies were already high, our model achieves marginal yet significant improvements, indicating potential for further enhancement. Specifically, the average accuracy on the 160x160 dataset increased by 0.37%, on the 120x120 dataset by 0.91%, and on the 80x80 dataset by 0.62%.

**Table 8:** Performance comparison of our proposed MCAM model with the previous state-of-the-art hybrid models from competitive studies on the GasHisSDB dataset. The best-achieved results are in bold. [Values in %].

**Model**	**Model components**	**Accuracy (%)**		
		**160x160**	**120x120**	**80x80**
Ensemble-WA5^41^	EfficientNetB0 + EfficientNetB1+ DenseNet121 + DenseNet169 + MobileNet (unweighted averaging)	99.20	98.68	97.72
Ensemble-UA5^41^	EfficientNetB0 + EfficientNetB1+ DenseNet121 + DenseNet169 + MobileNetV2 (weighted averaging)	99.16	98.69	97.69
Ensemble-MV5^41^	EfficientNetB0 + EfficientNetB1+ DenseNet121 + DenseNet169 + MobileNetV2 (weighted averaging)	99.16	98.69	97.69
Hybrid-DL^113^	EfficientNetV2B0 + CatBoost	93.99	93.18	89.72
Alexnet/ELM/AGTO^148^	AlexNet + Extreme Learning Machine + Dynamic Gorilla Troops Optimizer	whole	dataset	96.22
SVM^114^	Support Vector Machine with feature fusion	95.03	85.82	60.31
Random Forest^114^	Random Forest with feature fusion	92.26	89.56	78.44
**proposed MCAM**	Inception-V3 + VGG-16 + Xception (highest weighted voting)	**99.57**	**99.60**	**98.31**

**MCAM framework with and without TL:** To analyze the impact of TL on the experiment’s efficacy, we did a comparison analysis involving a model that contains TL and another that works without TL throughout the retraining phase. The findings of these experiments are presented in Table 9. Within the abnormal class, without TL, the MCAM model attained F1 scores of 98.11%, specificities of 98.12%, and sensitivities of 98.48%. In contrast, the inclusion of TL led to enhanced assessment measures, with values of 98.97% for sensitivity, 99.20% for specificity, and 98.91% for F1, implying enhancements of 0.49%, 0.08%, and 0.8%, respectively. However, within the abnormal category, in the absence of TL, the MCAM model recorded sensitivities, specificities, and F1 scores at 98.00%, 98.12%, and 98.21%, respectively. Conversely, when TL was integrated, considerable gains in assessment measures were detected, with values of 98.80% for sensitivity, 98.91% for specificity, and 99.00% for F1, suggesting enhancements of 0.80%, 0.79%, and 0.79%, respectively. The average accuracy for unfreeze and freeze layers is calculated as 98.25% and 98.87%, respectively, which means it is 0.62% higher than the model without TL. In short, the MCAM model with TL we proposed performs better than the model without TL.

**Table 9:** Model performance comparison with and without TL by freezing/unfreezing the network layer. [Values in %].

Class	Layers	Sens	Spec	F1	Pre	Avg. Acc
Normal	Unfreeze	98.00	98.12	98.21	97.22	98.25
	Freeze	98.80	98.91	99.00	98.24	98.87
Abnormal	Unfreeze	98.48	98.12	98.11	98.02	98.25
	Freeze	98.97	99.20	98.91	98.60	98.87

**Ensemble model without attention mechanism:** In our attempt to examine the utility of the attention mechanism module within the experiment, we opted to replace the MGIC, SIC, and MSIC with conventional models, especially Inception-V3, VGG-16, and Xception, forming an ensemble model. Compared with the outcomes from our suggested MCAM framework, the results produced from this ensemble model are generated from the averaging of data across three randomized experiments, as displayed in Table 10. Within the abnormal category, the ensemble model displayed sensitivity, specificity, and F1 values of 99.00%, 98.14%, and 98.64%, respectively. In contrast, our MCAM model surpassed the ensemble model with sensitivity, specificity, and F1 values of 99.12%, 98.91%, and 98.82%, showing improvements of 0.12%, 0.77%, and 0.18%, respectively, when compared to the ensemble model’s stated values. In the normal category, the ensemble model reports 98.29%, 98.14%, and 98.45% of sensitivity, specificity, and F1, respectively. In contrast, our proposed MCAM model reports 98.04%, 98.89%, and 98.88% of sensitivity, specificity, and F1 values. While our model exhibits a 0.25% decrease in sensitivity compared to the ensemble model, it achieves a 0.43% improvement in the crucial evaluation metric, F1 score. The average accuracy of our model is also 0.57% higher compared to the ensemble model. These findings emphasize the positive influence of the MCAM framework with added attention mechanism in boosting accuracy and resilience compared to the ensemble model with traditional DL models.

**Table 10:** Model performance comparison with and without attention mechanism. [Values in %].

Class	Attention mechanism	Sens	Spec	F1	Pre	Avg. Acc
Normal	not involved	98.29	98.14	98.45	97.90	98.15
	involved	98.04	98.89	98.88	98.10	98.72
Abnormal	not involved	99.00	98.14	98.64	98.22	98.15
	involved	99.12	98.91	98.82	98.40	98.72

**Fig. 12:**
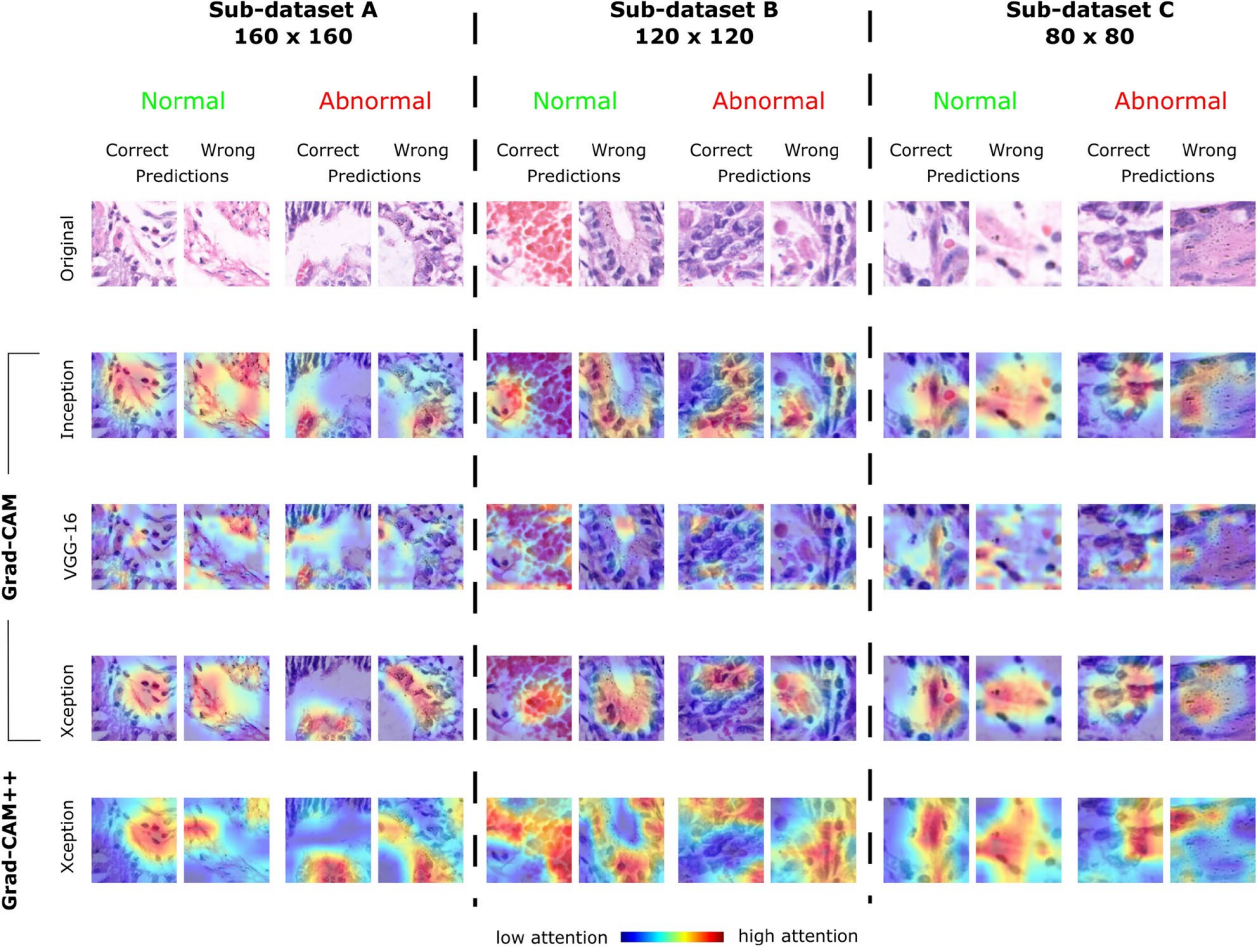
Visual explanations for three sub-datasets using deep networks Inception, VGG-16, and Xception models for normal and abnormal cases. The first row displays the original images, followed by three rows showcasing Grad-CAM results for each model and the final row illustrates Grad-CAM++ results for the Xception model.

**Performance analysis of the base models:** The Gradient-weighted Class Activation Mapping (Grad-CAM) maps shown in Fig. 12 highlight the regions of the input image that are most influential in determining the base model’s decision for classification. It generates a heatmap that is easier to interpret by using warmer colors to represent areas with greater impact. This makes it possible to comprehend the behavior of the model more fully, which helps with debugging and enhances model performance by pointing out and adjusting focus on features that aren’t relevant.

The sample images for normal and abnormal cases using the base models Inception, VGG-16, and Xception models across all sub-datasets are shown in Fig. 12. It is interesting to observe that each base model focused on different areas within the images for classification. Consequently, when an ensemble model was employed, it could analyze a broader set of features. This comprehensive analysis resulted in consistently superior performance to the individual base models. The accuracy of the base model improved as the resolution increased, moving from the low-resolution sub-dataset C (80x80) to the high-resolution sub-dataset A (160x160). This improvement is expected since higher-resolution images provide more detailed features for the model to analyze.

### Extended experiments

Here, we conduct a series of ablation experiments where we methodically break down the elements of our suggested model to identify their respective roles. Concurrently, we expand our assessment to include the HCRF dataset^149^ on gastric histopathology, offering a more comprehensive view of the model’s effectiveness on various datasets. Experiments on interchangeability are carried out on the three essential modules of the MCAM framework, which enhances our analysis and provides valuable insights into the flexibility and cooperation of these parts. This final section covers the computational time and experimental setup, thoroughly explaining the framework’s effectiveness. Furthermore, a competitive study with conventional DL models is provided, providing insightful information about the relative benefits and advantages of our proposed MCAM framework.

#### Ablation experiments

We systematically carried out a series of ablation tests using the experimental parameters provided in Section 4.1.3 to identify the precise contributions of the three channels within the MCAM framework. The results of these ablation tests are shown in Table 13, highlighting each channel’s unique importance and influence within the framework.

Firstly, the first row shows that the average accuracy when using only MGIC is only 0.18% lower than MCAM. However, it is noted that in the third ablation experiment, the sensitivity reported using only MGIC for the normal category is equal to the sensitivity for MCAM. Albeit in the abnormal category, the sensitivity is 0.02% higher than the MCAM. When MGIC is removed, the average accuracy is significantly reduced by 0.33% in the sixth row. Notably, these results highlight the essential role that the MGIC performs within the MCAM framework.

Secondly, in the context of the ablation experiment, especially in the third row, it is clear that the average accuracy obtained with MSIC alone is only 0.43% less than that of MCAM. In the second ablation experiment, it is noteworthy that the sensitivity for the abnormal category when MSIC is used exclusively outperforms MCAM by 0.21%. The average accuracy decreases by 0.16% in absence of MSIC channel, as shown in the fourth row. These results notably underscore the indispensable role played by MSIC within the comprehensive framework of MCAM.

Finally, the ablation experiment in the second row demonstrates that the average accuracy achieved with the SIC channel solely is 0.84% less than that of the MCAM framework. The average accuracy drops by 0.05% in the ablation experiment’s fifth row after removing the SIC. In contrast to the MCAM model, it was found that the first ablation experiment without using SIC had high sensitivity and F1 value for the abnormal category and high specificity for the normal category. Furthermore, in contrast to the MCAM framework, the sensitivity, specificity, and F1 were higher in the second ablation experiment without an SIC channel. The evaluation metrics for the SIC-less model in the third ablation experiment were lower than those for the MCAM. These experimental results show a limited but significant role for the SIC in the overall MCAM framework. It is readily apparent from the analysis of the ablation experiment that, in the broader MCAM framework, both the MGIC and MSIC channels play a crucial and distinctive role. The overall performance of the framework is noticeably worse in their absence. On the other hand, even though SIC plays a more minor role, it nevertheless enhances the framework’s functionality.

#### HCRF image classification

**Fig. 13:**
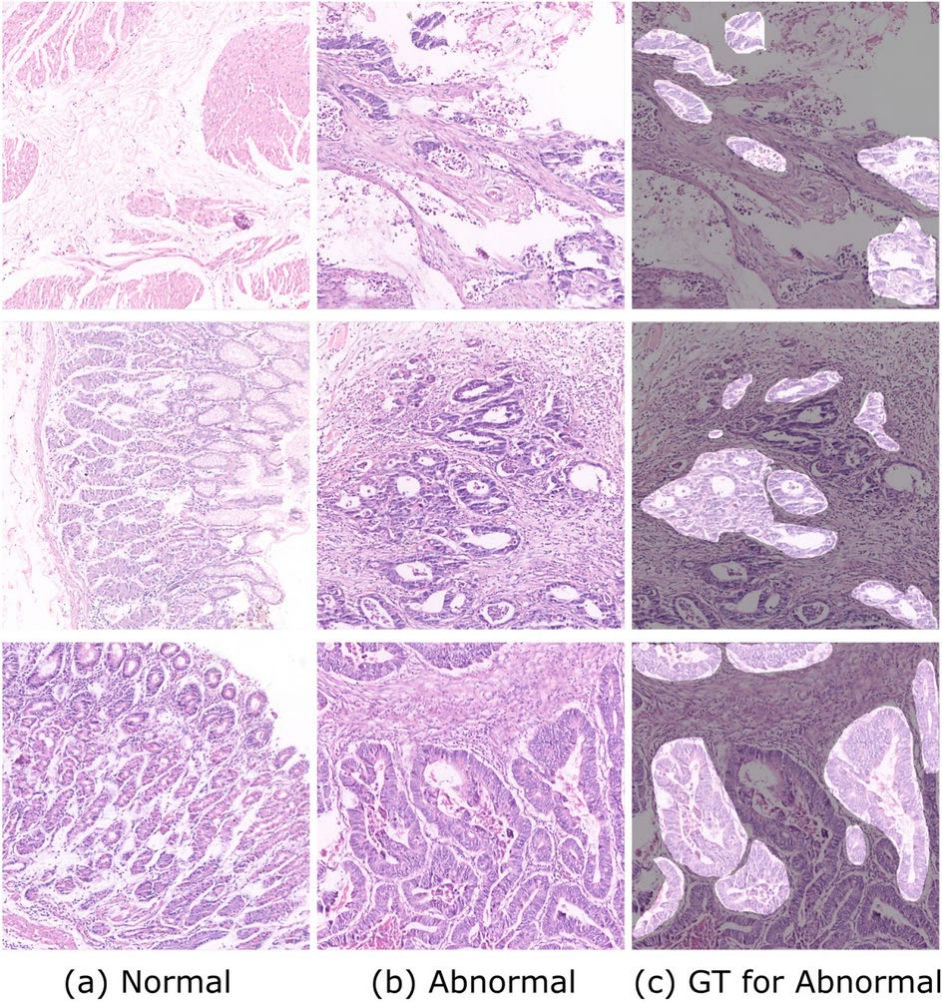
Examples of the stained gastric histopathological images from the HCRF dataset^149^. (**a**) shows the original images with normal condition (**b**) shows the original images having abnormal condition (**c**) represents the corresponding ground truth of the abnormal images provided in the dataset.

We conduct experiments on the publicly available H&E stained gastric histopathological image HCRF dataset in 20 magnification^150^, which is available in^151^ and shown in Fig. 13, to confirm that the MCAM framework has good generalization ability. The dataset images are in the format “*.tiff” or “*.png”. The dataset comprises 560 abnormal with corresponding GT and 140 normal images having resolution of 2048*x*2048 pixels. Dataset size is increased six times due to augmentation by flipping images horizontally and vertically and rotating 90, 180, and 270 degrees. Moreover, the images are cropped to 256 x 256 pixels due to the size of the gastric histopathology images being too large to process. Table 11 displays the data augmentation information. The HCRF dataset was selected for this study due to its diverse set of histopathology images captured under varying conditions, including differences in staining, illumination, and scanner resolutions. It includes images from multiple patient categories, ensuring variability in tissue morphology and pathological characteristics. This diversity enhances the model’s ability to generalize across different clinical settings, making it more robust to real-world variations.

**Fig. 14:**
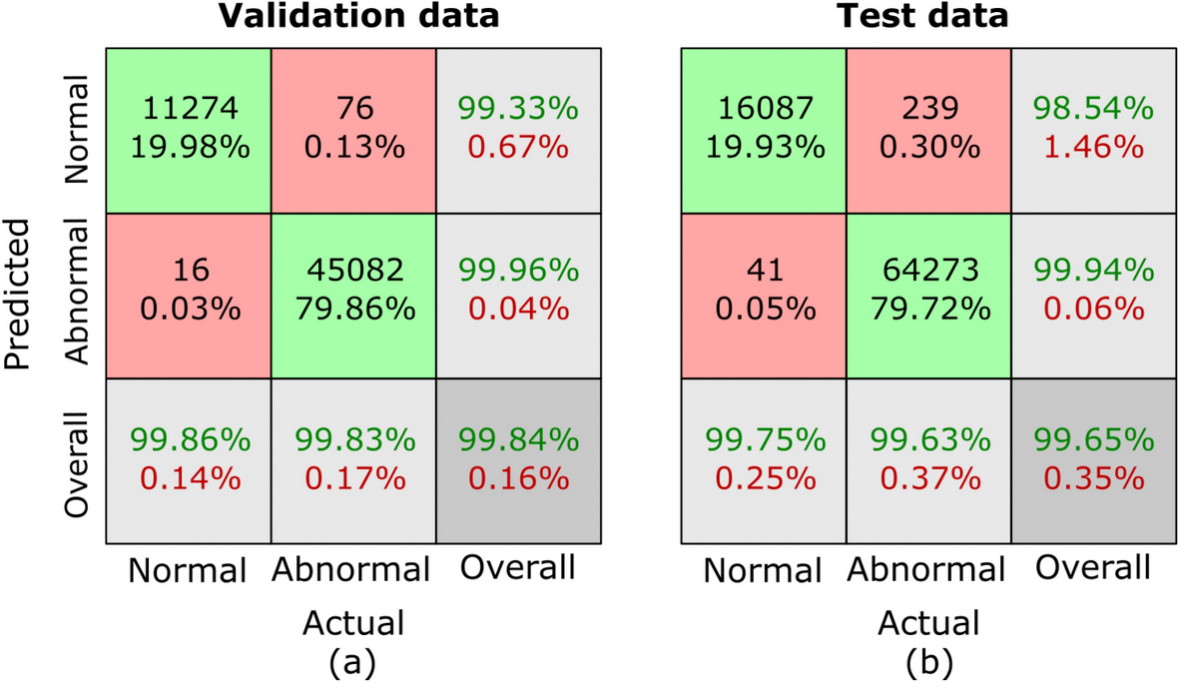
Confusion matrices for the HCRF dataset^149^ using proposed MCAM model. **(a)** shows the results on validation data **(b)** shows the results on testing data. The green blocks indicate the counts and percentages of true positive and true negative cases, while the red blocks represent false positive and false negative cases. In the last row, the first block shows sensitivity for normal cases and specificity for abnormal cases, the middle block shows sensitivity for abnormal cases and specificity for normal cases, and the last block represents the overall classification accuracy as a percentage. This visualization highlights the model’s consistent performance across all experiments.

The augmented dataset images are randomly divided into 70% training and 30% testing data. Further segmentation of the training data is performed, with the 70% training and 30% validation sets being randomly assigned. Table 12 shows the dataset distribution for training, validation, and testing. The proposed MCAM framework on the validation and testing set obtained an average accuracy of 99.87% and 99.64%, respectively. The confusion matrix on the validation and test sets are shown in Fig. 14. The percentage accuracy obtained on the validation and testing datasets are 99.84% and 99.65%. The calculated values of all the evaluation metrics are mentioned in Table 15. The accuracy achieved by our proposed MCAM model on validation and testing datasets is 99.84% and 99.65%. In the future, sophisticated segmentation techniques^23^ could show promising results in quantifying the abnormal region.

**Table 11:** HCRF data augmentation.

Number of images	Normal	Abnormal
Original	140	560
Augmented	53760	215040

**Table 12:** HCRF dataset distribution for training, validation, and testing.

Image class	Training	Validation	Testing
Normal	26342	11290	16128
Abnormal	105370	45158	64512
Sum	131712	56448	80640

**Table 13:** Performance evaluation of the proposed MCAM model in three randomized experiments on the testing dataset. The best-achieved values are in bold, while the second-highest values are underlined. The top values are individually highlighted in bold and underlined for normal and abnormal categories. ($\bullet$ indicates the used channels) [Values in %].

Channel	Class			1st Experiment			2nd Experiment			3rd Experiment			Avg.Acc
MGIC	SIC	MSIC		Sens	Spec	F1	Sens	Spec	F1	Sens	Spec	F1	
$\bullet$			N	98.96	98.14	98.36	98.24	98.57	98.69	99.70	98.67	98.78	
			A	98.52	98.27	98.11	98.67	98.42	98.57	98.94	98.24	98.77	98.70 ± 0.27
	$\bullet$		N	98.12	98.51	98.35	98.34	98.62	98.47	98.62	98.15	98.12	
			A	98.42	98.61	98.39	98.27	98.34	98.09	98.57	98.36	98.09	98.22 ± 0.09
		$\bullet$	N	98.32	98.21	98.47	98.42	98.57	98.31	98.25	98.47	98.51	
			A	98.22	98.34	98.27	98.48	98.25	98.31	98.44	98.15	98.25	98.41 ± 0.31
$\bullet$	$\bullet$		N	99.21	**99.67**	**99.78**	99.08	99.67	99.67	98.54	99.68	98.54	
			A	99.08	99.27	**99.67**	98.92	99.15	99.08	98.52	99.08	98.67	98.72 ± 0.27
$\bullet$		$\bullet$	N	99.42	99.58	99.39	98.78	99.02	98.72	99.25	99.68	98.76	
			A	**99.75**	99.28	99.63	98.80	99.08	98.72	98.79	99.09	98.90	99.80 ± 0.40
	$\bullet$	$\bullet$	N	98.68	98.32	98.57	98.78	98.24	98.35	98.92	98.47	98.05	
			A	98.54	98.25	98.47	98.62	98.34	98.67	98.91	98.15	98.15	98.60 ± 0.22
$\bullet$	$\bullet$	$\bullet$	N	99.70	99.48	99.58	98.32	98.69	98.67	**99.72**	99.47	99.07	
			A	99.72	99.29	99.50	98.21	98.72	98.47	98.92	**99.52**	99.18	**98.89** ± 0.26

#### Interchangeability experiments

We conduct an extended experiment under the constraints described in Section 4.1.3 to validate the interchangeability of the three modules inside the MCAM framework.

Since SimAM^134^ and CBAM^50^ both contribute weights to the spatial information of the VGG-16 model. Therefore, CBAM^50^ is used in SIC as a substitute for SimAM^134^. In the context of MGIC, SRM^47^, and ECA^46^ are alike to SE^45^ in that they allocate weights to channels of information. This enhances the Inception-V3 model’s capacity to extract global information at multiple scales. As a result, SRM^47^ and ECA^46^ are used in place of SE^45^. In MSIC, SRM and SE show similarities to ECA, increasing the Xception model’s ability to extract multi-scale global information. As a result, SE^45^ and SRM^47^ are used in place of ECA^46^. The results of the interchanged modules are listed in Table 14. The first row is the proposed MCAM model. However, the second row to the fifth is the interchanged tested attention mechanisms. The range of the substituted models’ average classification accuracy is 98.51% at its highest point to 98.10% at its lowest, indicating a variation of no more than 0.80% from the suggested MCAM model’s performance-a level of discrepancy that is well within an acceptable threshold. In short, there is possible interchangeability between the three channels in the proposed MCAM framework.

**Table 14:** Performance evaluation of the proposed MCAM model in three randomized experiments on testing datasets. The best-achieved values are in bold, while the second-highest values are underlined. The top values are individually highlighted in bold and underlined for normal and abnormal categories. [Values in %].

Channel			**Class**	1st Experiment			2nd Experiment			3rd Experiment			Avg. Acc
MGIC	SIC	MSIC		Sens	Spec	F1	Sens	Spec	F1	Sens	Spec	F1	
SE	SimAM	ECA	N	98.96	98.14	98.36	98.24	98.57	98.69	98.65	98.67	98.78	
			A	98.52	98.27	98.11	98.67	98.42	98.57	98.35	98.24	98.77	**98.84** ± 0.36
ECA	CBAM	SE	N	98.12	98.51	98.35	98.34	98.62	98.47	98.62	98.15	98.24	
			A	98.32	98.27	98.47	98.14	98.13	98.17	98.25	98.47	98.31	98.51 ± 0.11
SRM	CBAM	SRM	N	99.21	99.67	99.78	98.67	98.25	98.32	98.74	98.54	98.54	
			A	99.42	99.58	99.39	98.99	98.87	98.92	98.75	98.68	98.76	98.28 ± 0.31
SRM	CBAM	SE	N	99.21	**99.67**	**99.78**	98.67	98.25	98.32	98.62	98.54	98.69	
			A	99.08	99.27	**99.67**	98.80	98.32	98.58	98.79	98.99	98.90	98.10 ± 0.27
ECA	CBAM	SRM	N	**99.42**	99.58	99.39	98.87	98.72	99.02	98.75	98.68	98.76	
			A	**99.75**	**99.28**	99.63	98.80	98.82	98.58	98.79	98.99	98.90	98.39 ± 0.20

**Table 15:** Performance evaluation of the proposed MCAM model on validation and testing sets of HCRF dataset. [Values in %].

Class	Validation set					Testing set				
	Sens	Spec	F1	Pre	Acc.	Sesn	Spec	F1	Pre	Acc.
Normal	99.86	99.83	99.61	99.62		99.75	99.63	99.63	99.40	
Abnormal	99.83	99.86	99.47	99.60	99.84	99.63	99.75	99.47	99.20	99.65

#### Testing environment and computational time

A workstation having an Intel ®$\hbox {Core}^{\textrm{TM}}$ i7-8850H processor with a clock frequency of 3.60 GHz, 32 GB of RAM with an installed operating system of Windows 10 Professional, equipped with NVIDIA GeForce RTX 4060 8GB GPU is used to run the experiments. The workstation was configured with Python version 3.10.8, Torchvision 0.14.0, and Pytorch 1.13.0. The proposed MCAM model takes 1.17 hours or 4212 seconds in training.

## Discussion

Recently, DL models have become ever more significant in the field of medical diagnosis due to their dynamic advancement. In particular, categorizing histopathological images related to GC has become crucial for promptly identifying and avoiding diseases. To classify GasHisSDB, this paper presents and utilizes the MCAM framework, producing notable and efficient results. The proposed model is a modest yet pivotal stride toward advancing the automated diagnosis of GC.

Medical images are larger than conventional images and pose a unique challenge due to the non-uniform distribution of focused attention regions within the same class. For this peculiarity to be effectively analyzed, specialized approaches are frequently needed. Although highly effective, traditional CNN models tend to overcommit computational resources to edge information extraction because they mainly depend on convolutional kernels. This overemphasis on edges might not align with the subtle qualities of medical images, which necessitate a deeper comprehension of intricate patterns and structures. Consequently, it becomes necessary to implement alternative strategies, like integrating attention mechanisms, to guarantee that the computational resources of the model are optimally distributed among different relevant features. The combined framework addresses these particular issues, improving the models’ capacity to capture spatial details and multi-scale information while emphasizing the significance of modifying conventional techniques to meet the particular needs of medical image analysis.

One relevant aspect is that medical images are inherently complex, with complex anatomical structures and subtle variations that require the analytical models to be extremely sensitive. Furthermore, these models’ interpretability becomes critical in the medical domain, where obtaining high accuracy is not as important as comprehending the reasoning behind predictions. Another critical component is addressing issues with limited labeled data, a common problem in the medical field. Effective TL and data augmentation techniques can reduce this difficulty.

Our method incorporates an attention mechanism and a multichannel strategy to overcome this limitation. This combined framework aims to extract multi-scale information more easily by utilizing the benefits of a wide range of channels and attention mechanisms. By doing this, our model provides a more sophisticated and practical solution for image analysis in medical diagnostics, addressing the difficulties brought on by the special qualities of medical images. The VGG-16, Inception-V3, and Xception models are well known for their exceptional ability to extract essential data, such as multi-scale local features, multi-scale global information, and spatial details. Apart from their widely recognized ability to extract spatial details and multi-scale local and global attributes, the Xception, Inception-V3, and VGG-16 models provide a range of additional benefits. These models perform exceptionally well in TL, using their extensive pre-training on large datasets to show efficacy in situations with sparsely labeled data. Additionally, their architectures make it easier to extract robust hierarchical features, which is useful for tasks involving complex patterns and capturing both low-level and high-level representations. Because VGG-16, Inception-V3, and Xception have different architectural approaches, researchers and practitioners can select a model that best fits the demands of their particular tasks. These models have proven versatile beyond computer vision, finding use in various fields, including feature extraction, object detection, and image classification. The research and practitioner communities have widely adopted and supported these models, which has resulted in a wealth of resources, pre-trained models, and fine-tuning strategies that streamline the development and implementation process. Furthermore, these models’ scalability allows for modifications to meet particular tasks’ particular requirements or datasets’ complexity and size. Their tiered architecture also facilitates interpretability, a better understanding of the models’ decision-making processes and provides insights into the hierarchical features learned during training.

Fascinatingly, these models become much more effective when attention mechanisms like SimAM, SE, and ECA are included; this significantly improves recognition accuracy. The synergistic integration of these attention mechanisms provides a complementary ally to the inherent strengths of the base models. This integration highlights the models’ expertise and represents a sophisticated method of information extraction. Combining the attention mechanisms of SimAM, SE, and ECA provides a model demonstrating how to extract information more thoroughly and accurately, producing a noticeable improvement in recognition performance. The proposed MCAM framework uses three different channels (SIC, MSIC, and MGIC) to support the depth of information extraction and guarantee the complementarity of the learned insights. Meanwhile, three attention mechanisms are implemented to enhance the model’s depth further and protect the extracted data’s accuracy in each assigned channel. This combined method strengthens the width and depth classification performance and creates a subtle synergy between the channels and attention mechanisms. Essentially, the choice of the previously mentioned models forms the basis for building the overall MCAM model, which results in a novel framework that best utilizes width and depth considerations for improved classification abilities.

To enable a comprehensive comparison of the proposed methodologies with various traditional DL models, Table 16 presents an overview of the model parameters and training times. First, the suggested MCAM model performs very well, demonstrating a significant improvement in classification outcomes compared to traditional automatic techniques that use interactions. Moreover, even though other model types like MLP and VT generally outperform traditional CNN models in standard tasks and have proven to be adept at extracting global information, it is worth noting that these models’ performance in this particular experiment was subpar because of overfitting problems. The experimental results validate the notion that the small size of the medical training set is a major cause of overfitting when used to train large or complex models. Interestingly, ViT and CaiT models, which have large model parameters, did not produce acceptable results. On the other hand, the DeiT and T2T-ViT showed excellent classification performance. Similar trends are seen in MLP models, where better performance against the limited medical training set is attained through careful model architecture selection that favors more compact designs. In short, some small-scale models are computationally demanding due to the complexities brought about by the computational complexity of network structures. On the other hand, the MCAM framework uses simple convolutional and AM blocks and uses three channels: SIC, MGIC, and MSIC. By effectively reducing computation time across the three channels through parallel training techniques, the MCAM framework’s training efficiency is highlighted, even when significant model parameters are used.

**Table 16:** The training time and model parameters are compared between the suggested methods and other conventional DL models.

Framework/Model	Size (MB)	Time(s)
VGG-16^84^	512	7060
Xception^86^	79.6	4015
InceptionResNet-V1^89^	30.8	3260
AlexNet^136^	217	1331
Inception-V3^87^	83.4	5340
DenseNet-121^85^	27.1	2860
ResNet-50^83^	90	4772
ResNeXt-50^88^	88	4564
BoTNet-50^98^	72.1	4772
ViT^92^	31.2	1502
CoaT^96^	21.0	3120
DeiT^94^	21.1	2566
CaiT^93^	460	6956
LeViT^97^	65.8	2943
T2T-ViT^95^	16.01	2792
gMLP^143^	73.2	6396
MLP-Mixer^144^	225	11284
ResMLP^145^	169	8943
**MCAM**	**613**	**4212**

DL models must be successfully incorporated into practical medical applications, calling for a sophisticated strategy beyond algorithmic aptitude. Domain-specific knowledge must be incorporated because it enables researchers and developers to customize models to the specifics of medical diagnostic and imaging procedures. This necessitates deeply comprehending pathological variations, anatomical structures, and medically specific imaging nuances. An essential component of this process is collaboration with medical professionals, which helps to close the knowledge gap between technical proficiency and clinical judgment. Involving pathologists, radiologists, and other medical specialists improves the annotations in the dataset. It guarantees that the model’s predictions make sense in the context of medicine, which improves the model’s clinical relevance and interoperability.

Implementing DL models in real-world medical settings depends on the availability and effective use of computational resources, making this a crucial factor to consider before moving forward. Due to their high-resolution scans and large datasets, medical images are inherently complex and require significant processing power for training and inference. The computational intensity of tasks is increased by the size and depth of state-of-the-art models like VGG-16, Inception-V3, and Xception, which present difficulties in environments with limited resources. For real-time applications, where quick and precise diagnosis is essential, strong hardware and software optimized for efficient operation are required. Moreover, there is a constant need for more processing power due to the ongoing advancement of DL architectures and the exploration of ever-more complex models. The ability of computational infrastructure to scale up or down is crucial when models transition from experimental to practical application. While cloud-based solutions and distributed computing frameworks can potentially alleviate resource limitations, other considerations such as data privacy, network latency, and cost add to the complexity. Developing optimized model architectures, utilizing hardware accelerators, and investigating edge computing options are essential to improving the effectiveness of DL applications in healthcare environments. Maintaining accessibility and practicality in various healthcare settings while meeting the demanding requirements of medical workflows requires balancing computational power, energy efficiency, and real-time performance. Consequently, to fully realize the potential of DL models in transforming patient care and medical diagnostics, an integrated approach is required to address the impact of computational resources.

The proposed framework, incorporating three attention-based channels, alongside CNNs, increases computational complexity. Although transfer learning reduces the burden of training from scratch, fine-tuning still requires high-performance GPUs. This resource requirement may limit the models’ deployment in a resource-constrained clinical setting. The deployment of the model in resource-constrained clinical settings can require optimization techniques such as model pruning, quantization, and knowledge distillation. Furthermore, while the framework has been validated in two publicly available datasets, histopathological images vary between clinical settings due to differences in staining protocols, scanner resolutions, and patient demographics. Ensuring robustness across diverse environments requires domain adaptation techniques and testing on multi-center datasets. The Grad-CAM visualization provided results in interpretability; however, clinicians may require more detailed reasoning. Another limitation is the potential class imbalance in rare gastric cancer subtypes, which could introduce bias. Lastly, for real-world adoption, the model must support real-time processing and offer a user-friendly interface for pathologists. Future efforts will focus on optimizing inference speed and integrating the framework into digital pathology workflows to ensure seamless clinical implementation.

## Conclusion and future work

This study proposes a novel MCAM framework for GC detection using AMs with TL in histopathological images. The proposed MCAM framework uses a variety of AMs to facilitate automatic learning, showing notable improvements in GC detection over conventional DL models. The evaluation metrics, which were acquired through extensive testing, confirm the MCAM approach’s efficacy. In addition, three extensive sets of experiments are carried out: ablation experiments clarify the unique functions of every channel in the proposed model; interchangeability experiments confirm channels’ feasibility and interchangeability; and experiments on the HCRF dataset^149^ demonstrate the MCAM framework’s generalization abilities. Together, these results highlight the suggested framework’s encouraging potential as a reliable and flexible tool for precisely identifying GC in histopathological images.

Our strategy fills essential gaps in current methods while offering a more interpretable and sophisticated deep-learning framework, improving the field of GC classification. Our model successfully captures fine-grained cellular features and more general tissue-level patterns by integrating multiscale feature extraction and attention methods. This provides pathologists with simple, interpretable visual indicators and improves the accuracy of cancer vs. non-cancer differentiation. Furthermore, the robustness and flexibility of the model to actual clinical situations are guaranteed by our thorough validation of another dataset, including external cohorts. By doing this, our work improves the accuracy and usefulness of AI-driven diagnosis and lays the groundwork for a smoother transition of these tools into clinical practice, ultimately contributing to better patient outcomes.

The findings of this study have significant clinical implications, as the proposed MCAM framework provides an interpretable and highly accurate approach for GC classification using histopathology images. By enhancing feature extraction and integrating attention-based visual explanations, the framework can assist pathologists in reducing diagnostic errors. The ability to accurately distinguish between normal and abnormal cases, even in lower-resolution images, suggests that the framework could be integrated into digital pathology workflows, supporting early cancer detection and treatment planning. Additionally, the model’s robust performance across different datasets highlights its potential for real-world deployment in multi-center clinical settings.

Future directions in DL-based medical image analysis research are promising and highlight the ongoing pursuit of improved capabilities and responsible applications. One prominent area of focus is novel architectures, specifically the investigation of architectures adapted to particular medical imaging modalities and pathologies. Tailored models can potentially optimize the extraction of clinically relevant information by utilizing insights specific to a given domain. A more thorough understanding of medical conditions may also result from exploring the integration of multi-modal information, such as merging imaging data with genomics, patient records, or other contextual data. Another area of research is optimization techniques, where scientists are trying to find a way to balance computational efficiency and model complexity. DL models could be more easily integrated into point-of-care settings if techniques were developed that guarantee quick and accurate inference on various hardware configurations, including edge devices. Additionally, improving interpretability and explainability frameworks is essential to increasing healthcare practitioners’ trust in these models. This entails creating techniques to draw attention to pertinent aspects consistent with clinical reasoning and enhancing the transparency of model predictions. Deploying DL models in medical contexts should be guided by ethical frameworks that should be further explored in future research. Ethical considerations remain paramount. This entails dealing with concerns of justice, accountability, and bias and ensuring that strong procedures for informed consent, data privacy, and regulatory compliance are in place. Responsible guidelines for integrating DL models into routine medical practices will largely be shaped by collaborative initiatives between technologists, medical professionals, ethicists, and regulatory bodies. Finally, our suggested approach focuses on cropped or representative sections of the full-slide photos. This patch-based approach effectively captures the necessary local and global contextual information for classification while being computationally efficient. However, applying the proposed method directly to whole-slide image remains an important future direction. In future work, we plan to extend the framework by incorporating whole-slide image-level processing, which could include techniques such as multiscale patch extraction, whole-slide image-level aggregation, and advanced attention mechanisms to validate the generalizability and robustness of the proposed model in handling whole slide images for real-world clinical applications. In summary, there is potential for future research in DL-based medical image analysis. Researchers can play a significant role in the ongoing evolution of these models by investigating novel architectures, improving optimization techniques, and developing ethical frameworks. By doing so, they can ensure that these models’ capabilities align with the complex nature of healthcare while adhering to ethical conduct and responsibility principles.

## Data Availability

The GasHisSDB dataset is openly available at https://gitee.com/neuhwm/GasHisSDB (accessed on 24 June 2023). The HCRF dataset is openly available at https://data.mendeley.com/datasets/thgf23xgy7/2 (accessed on 02 January 2024). These datasets do not contain personal or identifiable information about individuals and are fully compliant with General Data Protection Regulation standards. The code developed for the proposed methodology has been made publicly available to facilitate future replication and further advancements in the field. It can be accessed using the following link: https://github.com/zubairfarooqi/GHCS.
